# The Global Epidemiology and Contribution of Cannabis Use and Dependence to the Global Burden of Disease: Results from the GBD 2010 Study

**DOI:** 10.1371/journal.pone.0076635

**Published:** 2013-10-24

**Authors:** Louisa Degenhardt, Alize J. Ferrari, Bianca Calabria, Wayne D. Hall, Rosana E. Norman, John McGrath, Abraham D. Flaxman, Rebecca E. Engell, Greg D. Freedman, Harvey A. Whiteford, Theo Vos

**Affiliations:** 1 National Drug and Alcohol Research Centre, University of New South Wales, Sydney, New South Wales, Australia; 2 Melbourne School of Population and Global Health, University of Melbourne, Melbourne, Victoria, Australia; 3 Queensland Centre for Mental Health Research, Brisbane, Queensland, Australia; 4 School of Population Health, University of Queensland, Brisbane, Queensland, Australia; 5 University of Queensland Centre for Clinical Research, University of Queensland, Brisbane, Queensland, Australia; 6 National Addiction Centre, Kings College London, London, United Kingdom; 7 Queensland Children’s Medical Research Institute, University of Queensland, Brisbane, Queensland, Australia; 8 Queensland Brain Institute, University of Queensland, Brisbane, Queensland, Australia; 9 Institute for Health Metrics and Evaluation, University of Washington, Seattle, Washington, United States of America; University of California, Irvine, United States of America

## Abstract

**Aims:**

Estimate the prevalence of cannabis dependence and its contribution to the global burden of disease.

**Methods:**

Systematic reviews of epidemiological data on cannabis dependence (1990-2008) were conducted in line with PRISMA and meta-analysis of Observational Studies in Epidemiology (MOOSE) guidelines. Culling and data extraction followed protocols, with cross-checking and consistency checks. DisMod-MR, the latest version of generic disease modelling system, redesigned as a Bayesian meta-regression tool, imputed prevalence by age, year and sex for 187 countries and 21 regions. The disability weight associated with cannabis dependence was estimated through population surveys and multiplied by prevalence data to calculate the years of life lived with disability (YLDs) and disability-adjusted life years (DALYs). YLDs and DALYs attributed to regular cannabis use as a risk factor for schizophrenia were also estimated.

**Results:**

There were an estimated 13.1 million cannabis dependent people globally in 2010 (point prevalence0.19% (95% uncertainty: 0.17-0.21%)). Prevalence peaked between 20-24 yrs, was higher in males (0.23% (0.2-0.27%)) than females (0.14% (0.12-0.16%)) and in high income regions. Cannabis dependence accounted for 2 million DALYs globally (0.08%; 0.05-0.12%) in 2010; a 22% increase in crude DALYs since 1990 largely due to population growth. Countries with statistically higher age-standardised DALY rates included the United States, Canada, Australia, New Zealand and Western European countries such as the United Kingdom; those with lower DALY rates were from Sub-Saharan Africa-West and Latin America. Regular cannabis use as a risk factor for schizophrenia accounted for an estimated 7,000 DALYs globally.

**Conclusion:**

Cannabis dependence is a disorder primarily experienced by young adults, especially in higher income countries. It has not been shown to increase mortality as opioid and other forms of illicit drug dependence do. Our estimates suggest that cannabis use as a risk factor for schizophrenia is not a major contributor to population-level disease burden.

## Introduction

Cannabis is a generic term for preparations (e.g. marijuana, hashish and hash oil) derived from the *cannabis sativa* plant[1]. The cannabis plant contains more than 60 unique cannabinoids. The one that is primarily responsible for the psychoactive effects that cannabis users typically seek is delta-9-tetrahydrocannabinol or THC[2-4], which is found in a resin on the flowering tops and upper leaves of the female plant. Most of the other cannabinoids are either inactive or only weakly active, although they may interact with THC[3,5] (2,4). THC acts upon a specific cannabinoid receptor (CB_1_) in the brain[6]. 

Cannabis is widely used in developed and developing countries[7,8]. Global patterns of cannabis use have been estimated by the United Nations Office on Drugs and Crime (UNODC)[9] but there has not been a systematic review of global, regional and country-level patterns of cannabis dependence and disease burden. This information is critical to inform policy and programming to prevent and treat this disorder.

The global burden of disease (GBD) framework was initiated by the World Bank report of 1993 [10] and uses information on mortality and disability associated with a given disease to estimate the years of life lost due to premature mortality (YLLs) and the years lived with disability (YLDs). YLLs and YLDs can be summed into disability adjusted life years (DALYs), an overall summary measure of disease burden. 

Previous GBD studies (GBD 1990 and its subsequent updates) significantly enhanced the global awareness of the burden of mental and substance use disorders[11-13] but did not include cannabis dependence. In 1990 the drug use disorder estimate was defined as ‘dysfunctional and harmful drug use’[11] and in 2002 ‘opioid dependence and harmful use and cocaine dependence’ were included as one group[12]. 

In 2002, the GBD “comparative risk assessment” (CRA) exercise[14] estimated the proportion of disease burden attributable to alcohol, tobacco, and illicit drug use[14]. Cannabis use was not included as a risk factor for any disease due to concerns about the quality of the evidence[15]. In the intervening years, there has been a steady increase in the quantity and quality of research on cannabis use and psychosis (or schizophrenia)[16-18]. Overall, these studies indicate that chance is an unlikely explanation of their association[16-18]. Recent reviews of prospective general population studies of associations between cannabis use and later psychosis[17,18] concluded that although control for confounding reduced the size of the association, there was an increased risk of psychotic outcomes in individuals who used cannabis, with the greatest risk among those who used cannabis most frequently. It is useful to distinguish two primary ways in which cannabis use could be a “cause” of psychosis[19]. The strongest form of causal link is that heavy cannabis use causes a psychosis that would not otherwise have occurred. A second hypothesis is that cannabis use is a contributory cause: it might precipitate psychosis in vulnerable individuals - that it is one factor among many (including genetic predisposition and other unknown causes) that act together to cause psychotic disorders. 

The evidence suggests that it is more likely than not that cannabis use precipitates schizophrenia in vulnerable persons[20-24]. This is consistent with other lines of evidence suggesting that there is a complex constellation of factors leading to the development of psychosis (the stress-diathesis model of schizophrenia) and with studies suggesting that gene-environment interactions may provide some explanation of the association [20]. It is also consistent with conflicting evidence to date on whether changes in cannabis use have been associated with changes in the incidence of psychotic disorders in the general population[21-23]. There is also some evidence that cannabis use is associated with increased likelihood of relapse to psychosis among those who have developed a psychotic disorder [25], although the quality of control for confounding in these studies is poor [25]. In some studies cannabis use has also been associated with a younger age of onset of psychosis [26], although control for confounding variables in these has also been poor.

### Aims

GBD 2010 updated the burden estimation methodology and estimated the burden of 291 diseases and 67 risk factors, by age, sex, 187 countries, and 21 world regions, for 1990, 2005 and 2010. It included both the direct burden attributable to cannabis dependence and the additional burden arising from cannabis dependence as a risk factor for other health outcomes. As part of the GBD 2010 study, we conducted systematic reviews of the literature to capture all the available data since 1990 on the prevalence and consequences of cannabis dependence. Other methodological improvements included: the use of a Bayesian meta-regression approach to model the epidemiological data and propagate uncertainty around final burden estimates; the quantification of disability for a more comprehensive list of health states using survey data from a more representative sample; and adjusting burden estimates for the effects of comorbid disorders[27,28].

This paper builds on our systematic reviews of the epidemiology of cannabis use and dependence[29-32], and the relationship between cannabis use and schizophrenia[24,30,33], and expands on previous analyses of the contribution of illicit drug use to the global burden of disease[27,28,34-36]. We expand considerably in this paper on what has been previously reported by conducting the first assessment of the global burden of cannabis dependence. We 1) outline the methodology used to estimate burden for this disorder specifically; 2) assemble data on the incidence and prevalence of cannabis use and dependence into a comprehensive disease model which adjusts for known sources of variability between studies; 3) investigate trends in the burden of cannabis dependence; and finally 4) investigate the model used in GBD 2010 to estimate the global burden of disease attributable to cannabis dependence as a risk factor for schizophrenia. 

## Methods

### Case definition

The case definition of cannabis dependence was based on the Diagnostic and statistical manual of mental disorders (DSM)[37] and International classification of diseases (ICD)[38] diagnostic criteria for cannabis dependence (DSM:304.30; ICD:F12.2).

### Systematic reviews

Systematic searches were conducted for studies published since 1990 to identify data sources for the prevalence, incidence, remission and all-cause excess mortality attributable to cannabis use and dependence. Full detail of these searches has been published elsewhere[30-32,39,40].The search strategy adhered to PRISMA guidelines[41] and used the methodology recommended by the Meta-analysis of Observational Studies in Epidemiology (MOOSE) group[42]. 

All extraction and quality assurance procedures were as recommended by the Strengthening the Reporting of Observational Studies in Epidemiology (STROBE) guidelines[43]. Studies were excluded if they did not contain primary data (e.g. review articles) or they contained data collected before 1990. We extracted estimates of annual incidence, current (including past-month) prevalence and period (past year, past-month) prevalence. Remission from cannabis dependence was defined as no longer fulfilling the diagnostic criteria for the disorder. For remission studies, we only included prospective studies that reported on a follow-up of at least three years [30]. 

An additional literature search identified *any* evidence of drug use within other regions. Evidence of *any use* was based on reports of derived estimates of use[44], sample estimates, drug-related treatments, drug seizures, drug-related arrests, or other qualitative information related to the use of illicit drugs. Sources of *any use* information include United Nations Office of Drugs and Crime reports, government reports, surveys, news reports and journal articles. We located evidence of cannabis use or dependence for almost all of the world’s population aged 15-64 years (201 countries/territories; 99.8% of the world’s population aged 15-64 years). There were estimates of the prevalence of cannabis use in 108 countries. We identified 13 studies (60 data points) from 4 GBD regions that reported on the prevalence of cannabis dependence. We found an additional 7 studies (57 data points) from 5 GBD regions reporting on weekly cannabis use and 80 studies (1313 data points) from 17 GBD regions reporting on past year cannabis use. We found only three general population cohort studies that reported on the incidence of cannabis use or dependence. We found no epidemiological evidence of elevated mortality risk attributable to cannabis dependence (for full details see[31,39]). The decision was therefore made not to assume elevated mortality for cannabis dependence. Details of the number and location of estimates are presented in Figure **S1** and Table **S1** **.**

### DisMod-MR modelling

We pooled these epidemiological estimates in a disease model that was judged to have face validity by the GBD 2010 Expert Group and additional experts in cannabis use epidemiology, in terms of age and sex patterns for the disease, differences in incidence or prevalence between regions, and changes in these parameters over time. For these estimates we used DisMod-MR[28,45], the latest application of an incidence-prevalence-mortality (IPM) mathematical model[46], re-designed as a Bayesian meta-regression tool for GBD 2010. The IPM model was implemented as a negative-binomial rate model to ensure internal consistency between separate estimates of prevalence, incidence, remission and excess-mortality. DisMod-MR was also used to predict epidemiological estimates for regions with no available data using country random intercepts, and prevalence estimates from elsewhere in the region and respective super-regional groupings (for details of GBD regions see Table **S2**). We preferred high quality, direct epidemiological estimates. In their absence, we used predicted estimates rather than exclude regions with no epidemiological data from GBD 2010 estimates[28,45]. 

There were two steps to use prevalence estimates of cannabis use to model cannabis dependence. Step one used DisMod-MR to model cannabis use and step two modelled cannabis dependence. Region-, sex- and year-specific cannabis use prevalence DisMod-MR output for the 20 to 44 age groups from step 1 were inserted into the cannabis dependence dataset. Cannabis use estimates were restricted to these age groups so as not to inflate the ratio of use to dependence and thereby make it difficult for DisMod-MR to derive a plausible fit to the cannabis dependence estimates. We used a ‘cannabis use’ study-level covariate to adjust estimates of cannabis use downwards towards its corresponding level if the studies produced survey estimates of cannabis dependence (see Text **S1** for an example of adjustment impacts). Values for incidence were set to zero before age 13 and after age 60 because the disorder is rare in children under 13 years and very rarely occurs for the first time in persons older than 60 years[47]. Incidence was derived using prevalence and remission and assuming no elevated mortality[31]. For examples of outputs see Figure **S2** **.**

### Disability weights

To estimate disability weights, a lay person description of cannabis dependence was formulated by the Expert Group as one of 222 lay descriptions that reflected the 291 diseases and their sequelae in GBD 2010. Full details of this process have been reported in Text **S1** and elsewhere [36,48]. The survey was completed by community samples in five countries (Bangladesh, Indonesia, Peru, the United Republic of Tanzania and the United States of America) and by respondents to an open-access internet survey. In both, a pair-wise comparison method, asking respondents to nominate the healthier of a pair of health states, was used to arrive at an estimate of the level of disability for each GBD health state. 

In keeping with the way in which GBD 2010 dealt with severity, survey data were used to adjust each disability weight for the severity of disorder. Full details of the process have been reported in Text **S1** and elsewhere [27,28]. After adjusting the DW for an estimated 51% (47%-54%) of cases who had no disability and accounting for comorbidity, the average disability weight for cannabis dependence was 0.162 (0.109 to 0.224). 

### Comorbidity adjustments

In order to correct YLDs for comorbidity, microsimulation methods were used to generate hypothetical populations by age, sex, year and country. Details are in Text S1and reported in full elsewhere in [27,28]. 

### Calculation of YLDs, YLLs and DALYs

GBD 2010 estimated ‘prevalent’ YLDs by multiplying prevalence estimates from DisMod-MR by the disability weight. DisMod-MR prevalence and burden estimates were stratified by sex, age, country and 21 GBD regions (see http://www.globalburden.com.au/docs/Regions.pdf and Table **S2** for country groupings), and for the years 1990, 2005, and 2010. 

We explored time trends in burden by breaking down the change in DALYs between 1990 and 2010 into changes that were attributable to: population growth, population ageing and sex structure, and changes in the epidemiology of cannabis dependence. As we found no evidence of excess deaths[31] (and therefore YLLs) directly attributable to cannabis dependence, the YLDs comprised all of DALY estimates[28].

### Comparative risk assessment (CRA): Cannabis use as a risk factor for schizophrenia

We reviewed existing literature on cannabis use as a risk factor for other health outcomes[24,31], as part of the CRA component of the study[34]. This review concluded that there was sufficient evidence to consider cannabis use as a risk factor for schizophrenia. We considered several ways in which cannabis and schizophrenia may be causally linked: 1) a model that assumed greater disorder severity among those using cannabis regularly who have already developed the disorder; 2) a model that assumed the association reflects earlier onset of schizophrenia among those who would have developed it anyway; 3) a model that assumed reduced remission from schizophrenia once it developed; and 4) a model that assumed an increased incidence of schizophrenia.

After consideration, approaches 3 and 4 were excluded from core GBD analyses because of the lack of data to systematically quantify the relationship across different studies while accounting for confounding variables. Approaches 1 and 2 were deemed more plausible on the basis of the literature[24] and so were included simultaneously in the modelling. 

Two systematic literature reviews were conducted on the global epidemiology of regular (weekly) cannabis use[29,32] and schizophrenia[49,50] respectively. Details are provided below. More information on the methodology is provided elsewhere [34]. 

### Data on regular (weekly or more frequent) cannabis use in the past year

We defined the exposure as weekly or more frequent cannabis use in the previous year because regular use is most consistently associated with this outcome. We found seven studies reporting prevalence of weekly or more frequent cannabis use in the past year, from 15 countries and five GBD world regions; and 80 studies on the prevalence of past-year cannabis use, from 82 countries and 17 GBD world regions[29,32].

The epidemiological data available for regular cannabis use were modelled using DisMod-MR. Estimates of prevalence were derived separately for 21 world regions, males and females, 5 year age groups. We assumed zero incidence and prevalence of regular cannabis use before age 13 as this led to the most plausible fit to the data. A study-level covariate was used to adjust estimates of past year cannabis use towards the estimates of weekly cannabis use. Prevalence from past year use were 3.79 (3.48-4.13) times higher than estimates of weekly cannabis use and were adjusted downwards accordingly.

### Modelling earlier age of onset of schizophrenia

The effect of cannabis use on schizophrenia was modelled via two pathways: the first by bringing forward the average age of onset in persons with no cannabis use, and the second by increasing the severity of schizophrenia. To account for the effects of the first pathway, we calculated the counterfactual average duration of schizophrenia across all ages under a scenario of no cannabis use, and compared this to the currently observed duration. To determine the counterfactual average duration of schizophrenia, we brought incident cases of schizophrenia who used cannabis forward by 2.70 (95% CIs: 1.96-3.43) years using results of a systematic meta-analysis [51]. 

We used the estimates of cannabis use by age (in single years), sex, country and year described above, assuming that the prevalence of regular cannabis use was the same among individuals with and without schizophrenia. Estimates of the number of incident cases and the corresponding duration of schizophrenia by age, sex, country and year were based on the DisMod-MR model for schizophrenia. The value of one minus the ratio of the counterfactual to the observed duration is an estimate of the population attributable fraction of schizophrenia due to the effect of regular cannabis use on age of onset.

### Modelling increased severity of schizophrenia

To calculate the burden associated with the second pathway of shifting severity, we used the odds ratio (OR) from Foti et al[52] of psychotic symptoms of 1.64 (95% CIs: 1.12-2.34) in people with schizophrenia who regularly use cannabis compared to those who do not. We converted the ORs to their RR equivalents based on the prevalence of exposure to regular cannabis use and the percent of time with psychosis (as opposed to residual state). The percent of time spent in acute psychosis was 63% (38%-82%) based on a meta-analysis of 6 studies covering 5 GBD world regions[49]. 

We used the estimates of cannabis use by age (in single years), sex, country and year described above and assumed that the prevalence of regular cannabis use was the same among individuals with and without schizophrenia. Estimates of the number of incident cases and the corresponding duration of schizophrenia by age, sex, country and year were based on the DisMod-MR model. We used the linear relationship between the estimated change in disability weight (based on the proportion of time spent in a psychotic state) and the prevalence of regular cannabis use to calculate the percent of schizophrenia disability attributable to regular cannabis use. Further detail on the modelling is found in Text **S2** **.**

Where we report comparisons of prevalence and DALYs by country or region we use age-standardised values using direct standardisation to the global standard population proposed by WHO in 2001 (http://www.who.int/healthinfo/paper31.pdf).

## Results

### Prevalence of cannabis dependence

**Figure 1:**
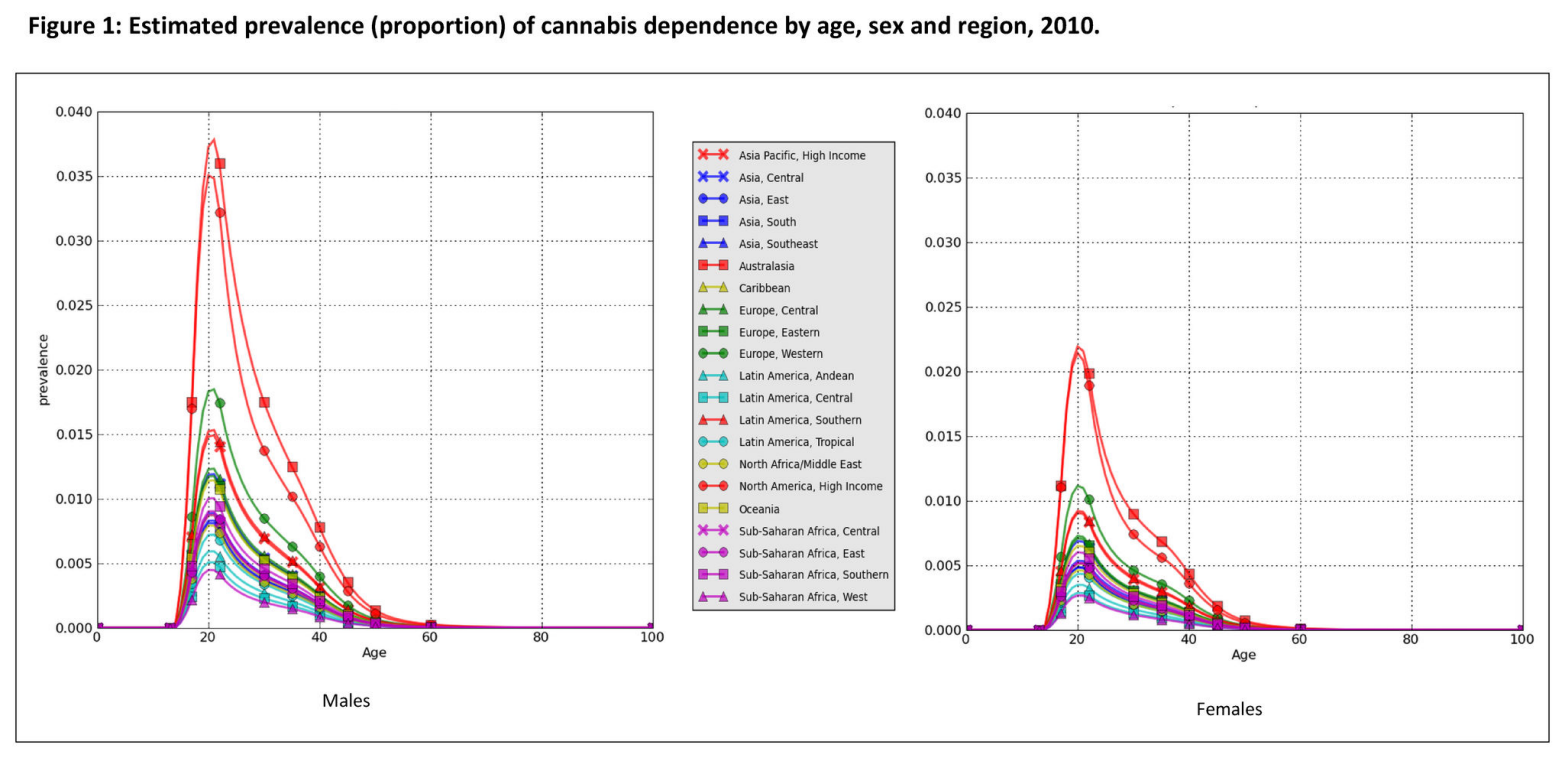
Estimated prevalence (proportion) of cannabis dependence by age, sex and region, 2010.

There were an estimated 11 million cases of cannabis dependence globally in 1990 and 13 million cases in 2010. These translated into pooled point prevalence estimates of 0.20% (95% uncertainty interval: 0.17-0.22%) in 1990 and 0.19% (0.17-0.21%) in 2010. These prevalence estimates were pooled across all regions and standardised by the 2010 global population age and sex. Figure 1 plots the point prevalence in 2010 by region, age and sex. 

Prevalence was higher in males (0.23% (0.2-0.27%)) than females (0.14% (0.12-0.16%)) producing a male: female sex ratio of 1.8 (1.7-1.9). Prevalence peaked in the 20-24 years age group at between 0.4% (0.3-0.6%) and 3.4% (2.8-4.2%) in males across regions, and between 0.2% (0.16-0.4%) and 1.9% (1.5-2.4%) in females. It decreased steadily with age thereafter. 

**Figure 2:**
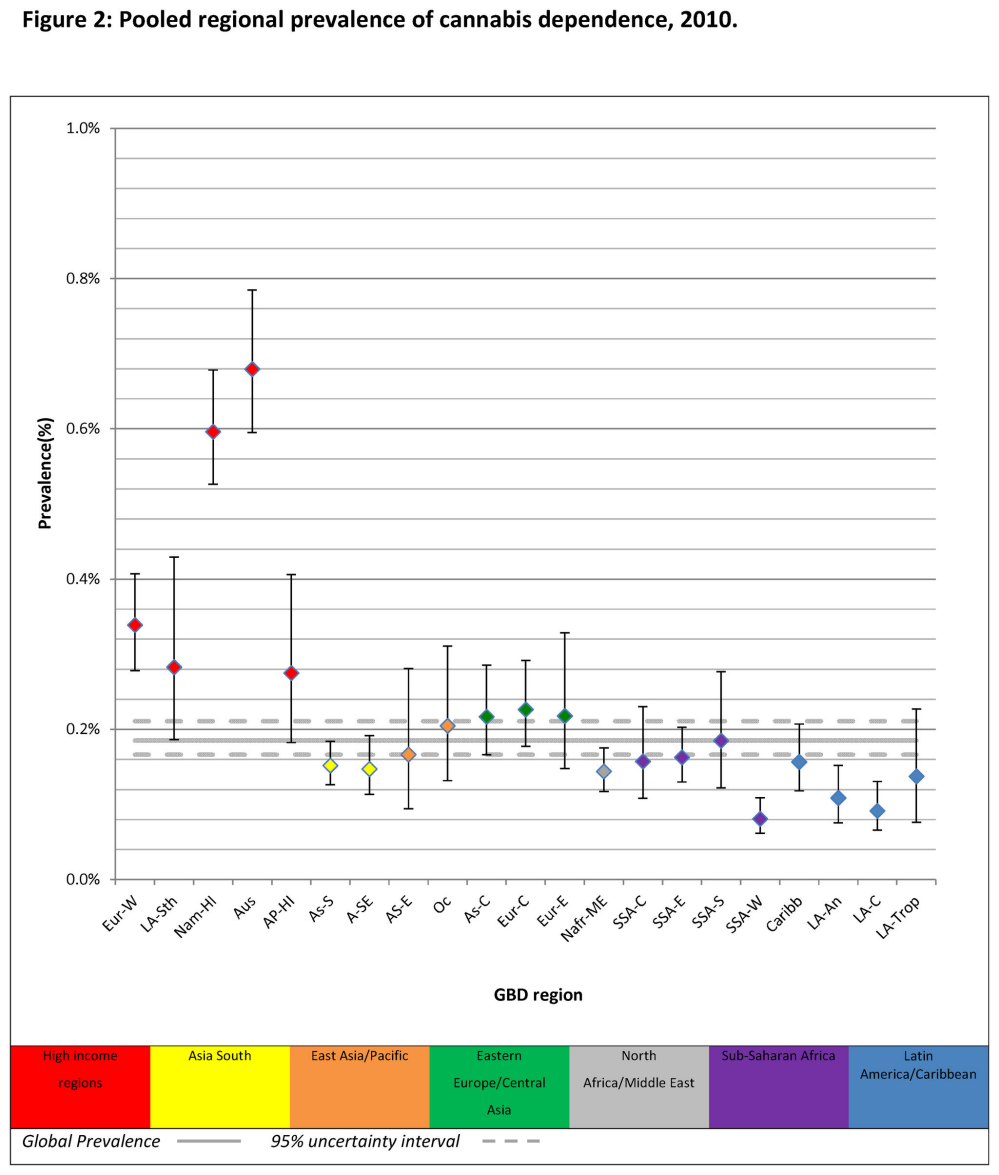
Pooled regional prevalence of cannabis dependence, 2010. *Note*. *Prevalence estimates were standardised by population age and sex; AP-HI: Asia Pacific, High Income, As-C: Asia Central, AS-E: Asia East, AS-S: Asia South, A-SE: Asia Southeast, Aus: Australasia, Caribb: Caribbean, Eur-C: Europe Central, Eur-E: Europe Eastern, Eur-W: Europe Western, LA-An: Latin America, Andean, LA-C: Latin America, Central, LA-Sth: Latin America, Southern, LA-Trop: Latin America, Tropical, Nafr-ME: North Africa/Middle East, Nam-HI: North America, High Income, Oc: Oceania, SSA-C: Sub-Saharan Africa, Central, SSA-E: Sub-Saharan Africa, East, SSA-S: Sub-Saharan Africa Southern, SSA-W: Sub-Saharan Africa, West.*

The regional variation in prevalence is summarised in Figure 2 and Table 1 (data on estimated prevalence and prevalence cases by region in 1990 is also reported in Table **S3**). Prevalence in high income regions was much higher than that in low to middle income regions and the global average. Cannabis dependence in Australasia (the region with the highest prevalence, 0.68%) was about 8 times higher than prevalence in Sub-Saharan Africa, West (the region with the lowest prevalence, 0.08%).Table **S4** also contains estimates of country-level prevalence of cannabis dependence. The wide and overlapping confidence intervals around some country and regional prevalence estimates indicate that not all variations in prevalence were statistically significant. 

**Table 1:** Estimated prevalence and number of cases of cannabis dependence in 2010, by sex and GBD region.

	Females			Males			Total		
	N	%	95%CI	N	%	95%CI	N	%	95%CI
**Asia Pacific, High Income**	147000	0.19	(0.15-0.26)	238000	0.26	(0.18-0.36)	385000	0.17	(0.09-0.28)
**Asia Central**	71000	0.14	(0.08-0.24)	125000	0.24	(0.18-0.33)	197000	0.28	(0.18-0.41)
**Asia East**	925000	0.15	(0.11-0.20)	1430000	0.26	(0.22-0.31)	2355000	0.22	(0.17-0.29)
**Asia South**	1001000	0.28	(0.18-0.42)	1601000	0.35	(0.32-0.39)	2602000	0.15	(0.13-0.18)
**Asia South East**	365000	0.23	(0.12-0.39)	610000	0.35	(0.22-0.54)	975000	0.15	(0.11-0.19)
**Australasia**	55000	0.20	(0.12-0.31)	98000	0.30	(0.25-0.36)	153000	0.68	(0.60-0.78)
**Caribbean**	26000	0.21	(0.13-0.35)	44000	0.15	(0.12-0.19)	69000	0.16	(0.12-0.21)
**Europe Central**	92000	0.24	(0.18-0.33)	155000	0.22	(0.20-0.25)	247000	0.23	(0.18-0.29)
**Europe Eastern**	160000	0.26	(0.22-0.31)	272000	0.28	(0.17-0.44)	432000	0.22	(0.15-0.33)
**Europe Western**	422000	0.26	(0.18-0.36)	710000	0.46	(0.41-0.53)	1132000	0.34	(0.28-0.41)
**Latin America, Andean**	23000	0.35	(0.32-0.39)	39000	0.27	(0.17-0.44)	62000	0.11	(0.08-0.15)
**Latin America, Central**	79000	0.35	(0.22-0.54)	142000	0.24	(0.17-0.35)	221000	0.09	(0.07-0.13)
**Latin America, Southern**	63000	0.30	(0.25-0.36)	105000	0.29	(0.22-0.37)	168000	0.28	(0.19-0.43)
**Latin America, Tropical**	107000	0.15	(0.12-0.19)	179000	0.15	(0.09-0.23)	287000	0.14	(0.08-0.23)
**North Africa/Middle East**	276000	0.22	(0.20-0.25)	447000	0.15	(0.11-0.20)	723000	0.14	(0.12-0.18)
**North America, High Income**	654000	0.28	(0.17-0.44)	1084000	0.14	(0.08-0.24)	1737000	0.60	(0.53-0.68)
**Oceania**	8000	0.46	(0.41-0.53)	13000	0.15	(0.11-0.20)	21000	0.20	(0.13-0.31)
**Sub-Saharan Africa Central**	56000	0.27	(0.17-0.44)	95000	0.19	(0.15-0.26)	151000	0.16	(0.11-0.23)
**Sub-Saharan Africa East**	215000	0.24	(0.17-0.35)	374000	0.28	(0.18-0.42)	589000	0.16	(0.13-0.20)
**Sub-Saharan Africa South**	56000	0.29	(0.22-0.37)	92000	0.23	(0.12-0.39)	148000	0.18	(0.12-0.28)
**Sub-Saharan Africa West**	104000	0.15	(0.09-0.23)	171000	0.20	(0.12-0.31)	275000	0.08	(0.06-0.11)
Global	**4906000**	**0.15**	**(0.11-0.20)**	**8023000**	**0.21**	**(0.13-0.35)**	**13,073,000**	**0.19%**	**(0.17-0.21)**

### Burden of cannabis dependence

Cannabis dependence accounted for 0.07% (0.05-0.1%) of global DALYs in 1990 and 0.08% (0.05—0.12%) in 2010. Although there was no change in the estimated contribution of cannabis dependence to global DALYs, there was an increase in crude DALYs across this period that was attributable to a 25% increase in population, a 6% decrease due to population ageing and a 1% increase in the prevalence rate of cannabis dependence. The net effect was a 22% increase in DALYs attributable to cannabis dependence between 1990 and 2010. 

**Figure 3:**
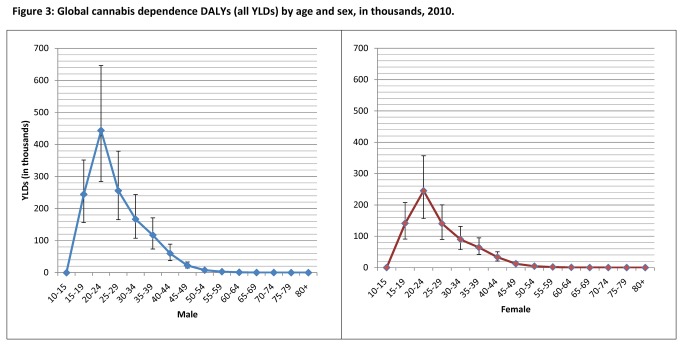
Global cannabis dependence DALYs (all YLDs) by age and sex, in thousands, 2010.

The remainder of the results focus on 2010 data. There were 2 million DALYs attributable to cannabis dependence in 2010, all YLDs. This accounted for 12.5% of YLDs attributable to illicit drug use and 0.27% (0.17-0.4%) of global all-cause YLDs. The majority of cannabis dependence YLDs occurred between 20 and 24 years (33.5% of cannabis dependence YLDs). In males this age group accounted for 64.3% of cannabis dependence YLDs. Figure 3 presents YLDs by age and sex.

**Figure 4:**
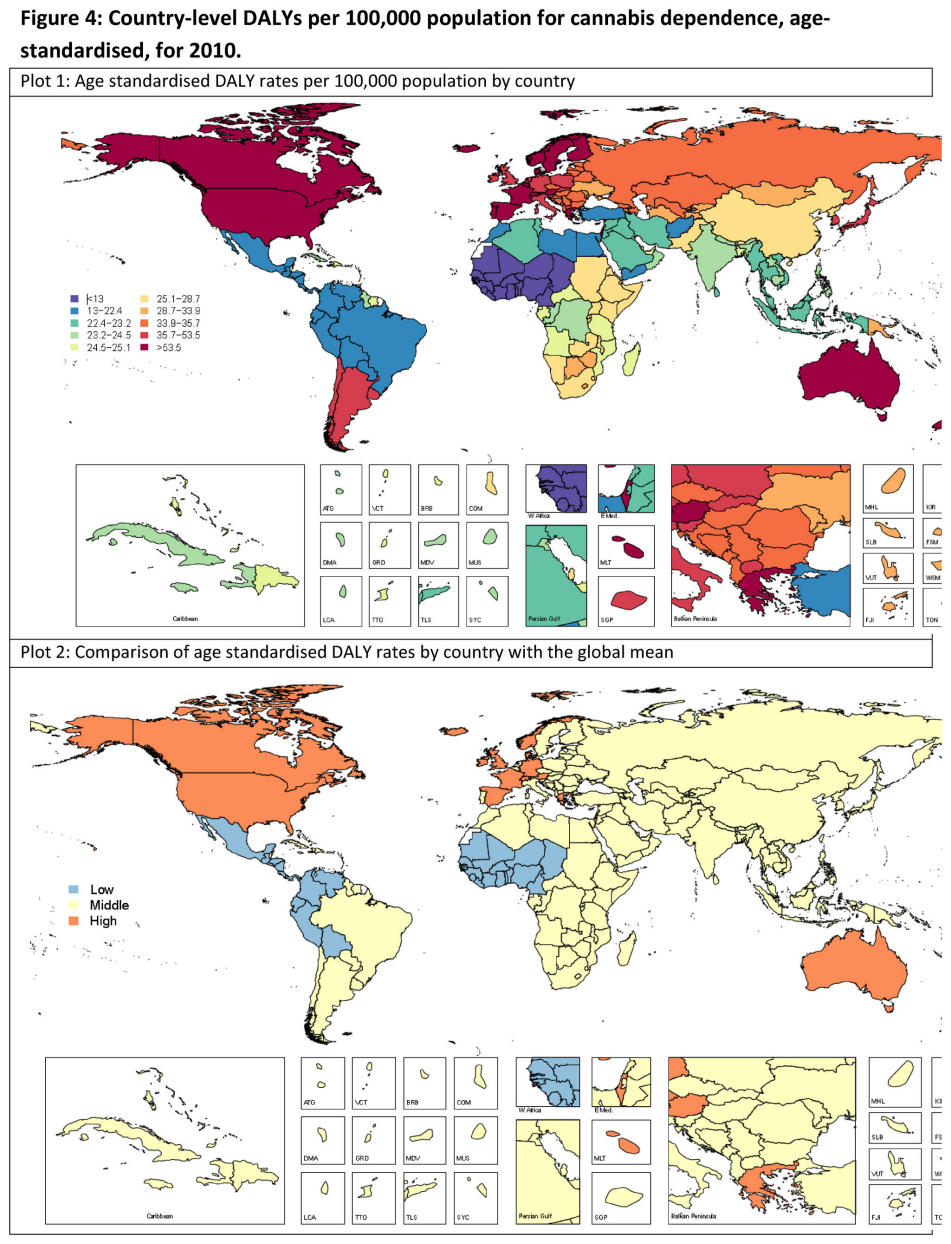
Country-level DALYs per 100,000 population for cannabis dependence, age-standardised, for 2010. Note. Low: shows countries with statistically lower DALY rates than global mean; Middle: Shows countries with DALY rates that are not statistically different to global mean; High: Shows countries with statistically higher DALY rates than global mean.

Table 2 presents the regional estimates of DALYs attributable to cannabis dependence; and Figure 4 presents age standardised DALY rates by country. There was substantial variation in DALYs between regions (table 2) and between countries (Figure 4a) but most of this variation occurred within wide and often overlapping bounds of uncertainty (Figure 4b). Table **S4** further details estimated country-level DALYs. As for estimated prevalence, there was considerable uncertainty around estimated rates for some countries and regions. The countries with statistically higher DALY rates than the global average were all from North America, high income, Australasia, and Europe Western regions. The countries with lower DALY rates than the global average were from Sub-Saharan Africa-West and Latin America Central and Andean. 

**Table 2:** Estimated DALYs for cannabis, by sex and GBD region, 2010.

	**Females**			**Males**			**Persons**			
	N	Rates per 100,000	95%CI	N	Rates Per 100,000	95%CI	N	Rates Per 100,000	95%CI	
	Asia Pacific, High Income	22000	24.4	(11.8-46.2)	39000	45.1	(21.5-82.5)	62000	34.5	(19.2-57.2)
	Asia Central	11000	27.7	(15.7-43.8)	20000	50.2	(29.2-81.0)	31000	38.7	(24.3-59.2)
	Asia East	135000	20.1	(7.9-45.2)	247000	34.1	(14.3-71.0)	382000	27.3	(13.4-50.5)
	Asia South	145000	18.6	(11.4-28.4)	270000	32.6	(19.7-48.7)	415000	25.8	(16.2-37.5)
Asia South East	56000	18.3	(10.4-29.8)	97000	32.0	(18.2-51.2)	153000	25.1	(15.8-37.7)
Australasia	8000	64.2	(41.5-93.7)	16000	122.5	(79.0-179.6)	24000	93.2	(61.7-134.2)
Caribbean	4000	18.1	(10.2-29.4)	7000	31.4	(17.6-49.5)	11000	24.7	(14.8-36.8)
Europe Central	14000	22.8	(13.6-35.8)	25000	43.9	(26.1-70.9)	39000	33.0	(20.5-50.1)
Europe Eastern	25000	22.9	(11.0-42.2)	43000	45.0	(22.3-78.6)	68000	33.1	(18.5-55.7)
Europe Western	64000	30.0	(18.4-44.6)	115000	56.5	(34.4-85.8)	179000	43.0	(27.0-62.3)
Latin America, Andean	4000	13.4	(6.3-23.0)	6000	23.0	(11.9-41.0)	10000	18.2	(10.2-29.8)
Latin America, Central	13000	11.2	(6.0-19.1)	22000	19.5	(10.5-32.7)	35000	15.3	(9.1-23.7)
Latin America, Southern	10000	31.0	(15.0-59.4)	17000	57.7	(28.6-106.7)	26000	44.1	(24.9-75.9)
Latin America, Tropical	17000	16.5	(6.5-34.5)	29000	29.4	(11.8-59.9)	46000	22.9	(11.4-41.0
North Africa/Middle East	40000	18.4	(11.4-28.6)	75000	33.0	(19.9-50.7)	115000	25.9	(16.2-37.7)
North America, High Income	98000	57.1	(37.0-82.1)	178000	106.4	(66.6-156.6)	276000	81.5	(53.6-116.6)
Oceania	1000	24.4	(11.1-47.0)	2000	41.4	(20.3-81.9)	3000	33.1	(17.4-57.6)
Sub-Saharan Africa Central	9000	17.7	(8.3-32.7)	14000	30.1	(14.5-57.0)	23000	23.9	(12.8-39.7)
Sub-Saharan Africa East	34000	18.8	(11.1-30.0)	58000	33.0	(19.9-51.7)	92000	25.9	(16.5-38.5)
Sub-Saharan Africa South	8000	23.6	(11.4-43.5)	15000	42.3	(20.9-78.8)	23000	32.8	(18.0-56.4)
Sub-Saharan Africa West	16000	9.6	(5.3-15.8)	27000	16.0	(9.2-27.0)	43000	12.8	(8.0-20.4)
**Global**	734000	21.5	(14.1-31.4)	1323000	38.1	(24.4-55.4)	2057000	29.9	(19.5-43.1)

**Figure 5:**
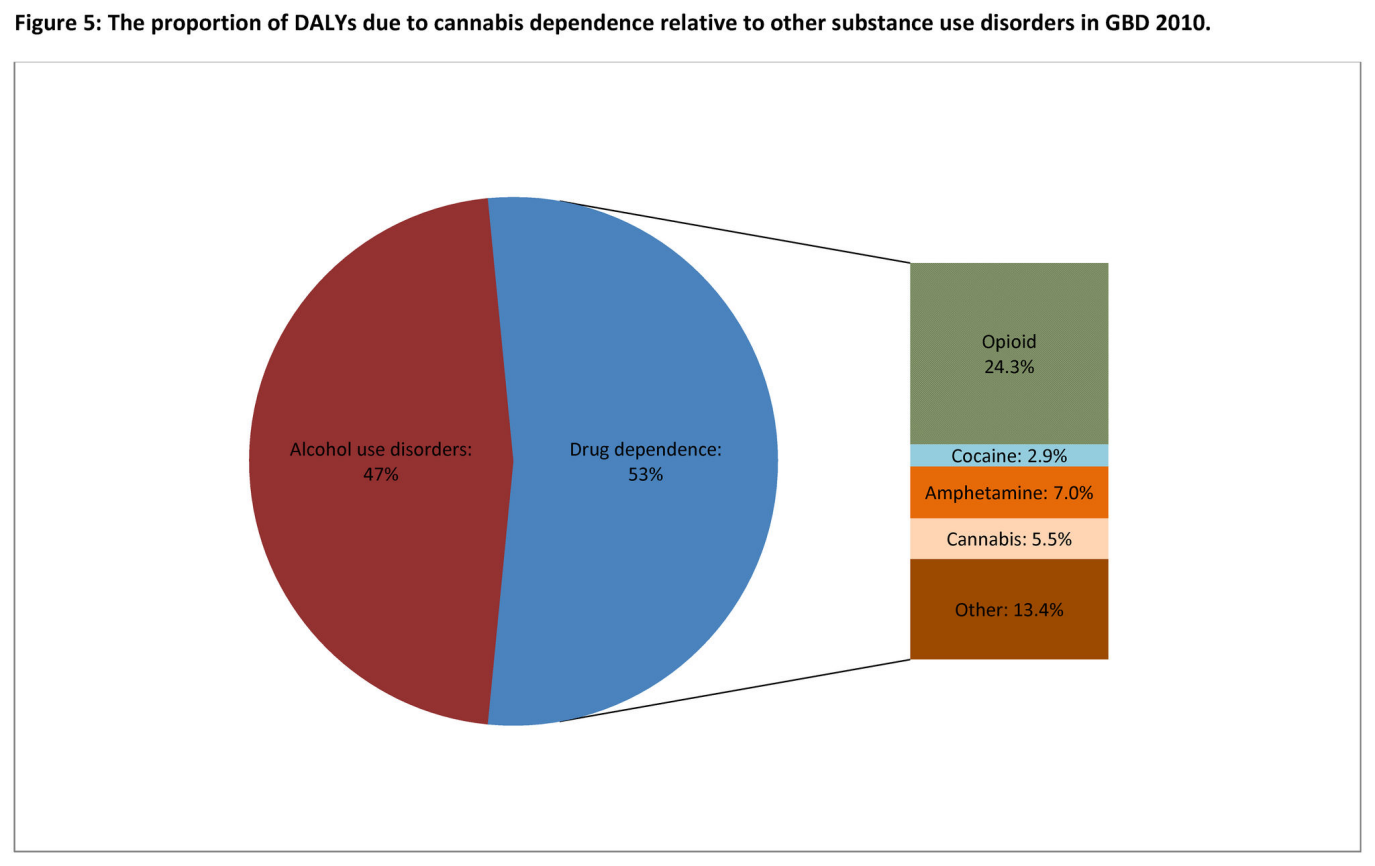
The proportion of DALYs due to cannabis dependence relative to other substance use disorders in GBD 2010. *Note*. *Alcohol use disorders included alcohol dependence and foetal alcohol syndrome; Other drugs: Burden attributable to illicit drug types other than cannabis, opioid, amphetamine and cocaine dependence were estimated under residual categories in GBD 2010*.

Figure 5 compares the burden of cannabis dependence to other substance use disorders in GBD 2010. Cannabis dependence was the only substance for which there were no attributable deaths and hence zero YLLs. Globally, it accounted for more DALYs than cocaine dependence, but fewer than the other drug use disorders. 

In high income regions such as Australasia, North America, high income and Europe Western where we had better quality epidemiological data available, the DALY rate (per 100,000) of cannabis dependence surpassed that attributable to amphetamine dependence (93.2 (61.7-134.2) vs. 58.5 (31.2-98.1) in Australasia; 43.03(27.0-62.3) vs. 34.5 (19.6-55.2) in Europe Western; and 81.5 (53.6-116.6) vs. 33.4 (18.4-52.8) in North America, high income).

### Additional burden attributable to cannabis use as a risk factor for schizophrenia

Regular cannabis use as a risk factor for schizophrenia accounted for 7,000 DALYs (3,000-13,000) or 0.04% (0.03-0.06%) of schizophrenia DALYs globally (Table 3). Overall, North America, high income was the region with highest number of attributable DALYs (2,000 DALYs). Males were responsible for over twice as many attributable schizophrenia DALYs (5,000 DALYs; 2,000-10,000) than females (2,000; 1,000-4,000); and attributable DALYs peaked between 25 and 30 years and decreased thereafter. When the additional burden attributable to cannabis use as a risk factor for schizophrenia was added to that of cannabis dependence, cannabis use still accounted for 0.08% of global DALYs in 2010.

**Table 3:** Estimated DALYs attributable to regular cannabis use as a risk factor for schizophrenia^**1**^, 2010.

	**Persons**			**Males**			**Females**		
	**Lower CI**	**Mean**	**Upper CI**	**Lower CI**	**Mean**	**Upper CI**	**Lower CI**	**Mean**	**Upper CI**
Attributable YLLs	-	**-**	-	-	**-**	-	-	**-**	-
Attributable YLDs	3,000	**7,000**	13,000	2,000	**5,000**	10,000	1,000	**2,000**	4,000
Attributable DALYs	3,000	**7,000**	13,000	2,000	**5,000**	10,000	1,000	**2,000**	4,000

Note. ^1^ Modelled with two effects; an earlier onset of schizophrenia among people using cannabis regularly; and increased time spent in the acute state of schizophrenia.

## Discussion

To our knowledge this is the first study to estimate global, regional, and country-level prevalence of cannabis dependence, and to estimate its contribution to the global burden of disease. An estimated 13 million people were cannabis dependent in 2010, an age and sex-standardised prevalence of 0.2% (0.17-0.22%). Prevalence was not estimated to have changed significantly from 1990, although increased population size produced an increase in the number of cases of cannabis dependence over the period. Levels of cannabis dependence were significantly higher in a number of high income countries including Australia, New Zealand, the United States, Canada, and a number of Western European countries include the United Kingdom. Cannabis dependence caused 2 million DALYs in 2010. DALY rates also varied considerably geographically, with the highest rates again in North America high income, Australasia and Western Europe. 

There is clearly scope to reduce the burden associated with cannabis dependence. The estimates presented in this paper are potentially useful for service planning at global, regional and country levels. Although cannabis use was estimated to be a smaller contributor to disease burden than alcohol or opioids, nonetheless some 2 million years lived with disability were attributed to the drug. Behavioural interventions are effective in the treatment of cannabis dependence[53,54], with cognitive behavioural therapy and contingency management showing the greatest promise. Public health campaigns may also be necessary to advise young people of the risks of developing dependence on cannabis because this risk may be underappreciated by many users. 

Based on the best available evidence, and models making reasonable assumptions, we found that only 0.04% of the DALYs attributed to schizophrenia were linked to regular (weekly or more frequent) cannabis use. Although epidemiological studies make a consistent case that early and/or heavy cannabis use is linked to a significantly increased risk of schizophrenia[24], the modest increase in risk and the low prevalence of schizophrenia mean that regular cannabis use accounts for only a very small proportion of the disability associated with schizophrenia. From a population health perspective, this raises doubt about the likely impact of preventing cannabis use on the incidence or prevalence of schizophrenia until further evidence finds that there is a causal relationship between regular cannabis use and the onset of new cases of psychotic illness [55].

### Limitations

We have made estimates based on the best available epidemiological data and used sophisticated modelling to incorporate a range of sources of uncertainty about the parameters used in our models. However, our reviews identified clear gaps in existing epidemiological data on cannabis dependence. A significant amount of research is needed to document even the most basic epidemiological parameters for cannabis dependence in most countries. Until such work is done, considerable uncertainty will remain around the exact size of global burden of disease that is attributable to cannabis and other illicit drugs. This is particularly the case for low income countries, where there is typically limited information on use occurring, even less on levels of use, and usually no data on prevalence of dependence. There is a clear imperative to better assess levels of dependent drug use in these countries whose populations may be experiencing higher levels of burden than were estimated here. 

Further, a range of potential health outcomes of cannabis use were not included in our estimates. We considered including suicide, cancer and accidental injuries but the evidence for a causal relationship for these outcomes was considered to be too weak to generate defensible global estimates[31].

We also note that we assumed that levels of cannabis use among people with schizophrenia were the same as the general population. This was a conservative assumption. There are data from clinical studies that very high levels of cannabis use are found among patients with schizophrenia[56]. However, a systematic review found that available data on the extent of this elevation were limited[56] to high income countries. It is also useful to be mindful of the impact of this assumption on the magnitude of the burden we are considering. This particular aspect is only relevant for the ‘increased severity’ analysis. The overall global attributable burden was only 7,000 DALYs. Even if levels of cannabis were several times higher among people living with schizophrenia, the extent to which we might be underestimating attributable burden would remain very small.

It is also important to acknowledge that the improved disability weights[36], involving surveys of the general population, have their limitations. As discussed elsewhere[36], it is unclear whether brief lay descriptions can accurately capture the complexity of disability due to disorders. There is also the possibility that considerations other than health status may have influenced respondents’ views of “which state was healthier” because it was hard to describe the disability due to cannabis use without mentioning it. Nonetheless, this study has made significant improvements in methods and in the transparency with which burden estimates have been made. 

### Conclusions

Cannabis dependence causes disability across the globe. It is a disorder primarily experienced by young adults, and our estimates suggest that burden is higher in high income countries. It has not been shown to increase mortality as opioid and other forms of illicit drug dependence do. Nonetheless, in some countries cannabis dependence produces more years lived with disability than drugs like amphetamines and cocaine, largely because rates of cannabis use are higher than for the stimulant drugs. Our estimates suggest that cannabis use as a risk factor for schizophrenia is not a major contributor to population level disease burden.

## Supporting Information

Figure S1 **Geographical representations of epidemiological data sources available for cannabis use and dependence.** *Note*. *The world map shows the number of studies from each region included in the DisMod-MR modelling*. *Some studies reported estimates for more than one region; P-Use: Number of studies reporting on prevalence of cannabis use; P-dep: number of studies reporting on prevalence of cannabis dependence; R: number of studies reporting on remission from cannabis dependence; We also found 3 studies reporting on the incidence of cannabis dependence which have not been summarised here as they were not included in the DisMod-MR modelling*. *They were inconsistent to the data available for prevalence and remission*.
(TIF)

Figure S2 **Cannabis use DisMod-MR prevalence output by region, age and sex in 2010.**
(TIF)

Table S1 **Summary of epidemiological data sources available for cannabis use and dependence.** Note. References of the included data sources have been presented elsewhere[29-32]; *Studies may have reported estimates for the overall age group, age specific estimates falling within the overall age group, or both.
(DOCX)

Table S2 **Countries located in regions and super regions as defined in GBD 2010.**
(DOCX)

Table S3 **Estimated prevalence and number of cases of cannabis dependence in 1990, by sex and GBD region.**
(DOCX)

Table S4 **Country-level estimates of cannabis dependence and DALYs per 100,000 due to cannabis dependence, sex and age-standardised, 2010.**
(DOCX)

Text S1 **Additional information on the estimation of burden for cannabis dependence in GBD 2010.**
(DOCX)

Text S2 **Comparative risk assessment: cannabis use as a risk factor for schizophrenia.**
(DOCX)
