# Correction: The Global Epidemiology and Contribution of Cannabis Use and Dependence to the Global Burden of Disease: Results from the GBD 2010 Study

**DOI:** 10.1371/journal.pone.0165221

**Published:** 2016-10-19

**Authors:** The PLOS ONE Staff

There are errors in the prevalence estimates reported in Table 1. Please see the corrected Table 1 here.

**Table 1:** Estimated prevalence and number of cases of cannabis dependence in 2010, by sex and GBD region.

	**Females**			**Males**			**Total**		
	N	%	95%CI	N	%	95%CI	N	%	95%CI
**Asia, Central**	73,000	0.16	(0.11–0.23)	125,000	0.28	(0.19–0.39)	198,000	0.22	(0.17–0.29)
**Asia, East**	853,000	0.12	(0.05–0.25)	1,547,000	0.21	(0.09–0.4)	2,400,000	0.17	(0.09–0.28)
**Asia Pacific, High Income**	141,000	0.2	(0.11–0.35)	248,000	0.34	(0.19–0.58)	389,000	0.28	(0.18–0.41)
**Asia, South**	935,000	0.11	(0.09–0.14)	1,718,000	0.19	(0.15–0.24)	2,653,000	0.15	(0.13–0.18)
**Asia, Southeast**	359,000	0.11	(0.08–0.16)	616,000	0.19	(0.13–0.26)	975,000	0.15	(0.11–0.19)
**Australasia**	54,000	0.48	(0.4–0.58)	100,000	0.87	(0.72–1.04)	154,000	0.68	(0.6–0.79)
**Caribbean**	26,000	0.12	(0.08–0.17)	44,000	0.2	(0.14–0.29)	69,000	0.16	(0.12–0.21)
**Europe, Central**	89,000	0.16	(0.12–0.23)	161,000	0.29	(0.21–0.4)	250,000	0.23	(0.18–0.29)
**Europe, Eastern**	163,000	0.16	(0.09–0.28)	273,000	0.27	(0.16–0.46)	436,000	0.22	(0.15–0.33)
**Europe, Western**	410,000	0.25	(0.19–0.33)	733,000	0.43	(0.33–0.56)	1,143,000	0.34	(0.28–0.41)
**Latin America, Andean**	23,000	0.08	(0.05–0.13)	39,000	0.14	(0.08–0.22)	62,000	0.11	(0.08–0.15)
**Latin America, Central**	83,000	0.07	(0.04–0.11)	140,000	0.12	(0.07–0.18)	223,000	0.09	(0.07–0.13)
**Latin America, Southern**	61,000	0.21	(0.12–0.35)	108,000	0.36	(0.2–0.61)	169,000	0.28	(0.19–0.43)
**Latin America, Tropical**	108,000	0.1	(0.04–0.21)	186,000	0.17	(0.08–0.33)	294,000	0.14	(0.08–0.23)
**North Africa/Middle East**	259,000	0.1	(0.08–0.14)	478,000	0.18	(0.14–0.24)	736,000	0.14	(0.12–0.18)
**North America, High Income**	629,000	0.44	(0.36–0.52)	1,127,000	0.75	(0.63–0.9)	1,755,000	0.6	(0.53–0.68)
**Oceania**	8,000	0.15	(0.08–0.27)	13,000	0.25	(0.15–0.45)	21,000	0.2	(0.13–0.31)
**Sub-Saharan Africa, Central**	56,000	0.12	(0.07–0.19)	93,000	0.2	(0.11–0.34)	149,000	0.16	(0.11–0.23)
**Sub-Saharan Africa, East**	217,000	0.12	(0.09–0.16)	373,000	0.21	(0.15–0.28)	590,000	0.16	(0.13–0.2)
**Sub-Saharan Africa, Southern**	54,000	0.14	(0.08–0.23)	94,000	0.23	(0.13–0.38)	149,000	0.18	(0.12–0.28)
**Sub-Saharan Africa, West**	103,000	0.06	(0.04–0.09)	173,000	0.1	(0.07–0.15)	276,000	0.08	(0.06–0.11)
**Global**	**4,704,000**	**0.14**	**(0.12–0.16)**	**8,389,000**	**0.23**	**(0.20–0.27)**	**13,091,000**	**0.19**	**(0.17–0.21)**

There are errors in Table 2. Rows 3 to 6 are misaligned. The publisher apologizes for the error. Please see the correct Table 2 here.

**Table 2:** Estimated DALYs for cannabis, by sex and GBD region, 2010.

	Females			Males			Persons		
	N	Ratesper 100,000	95%CI	N	RatesPer 100,000	95%CI	N	RatesPer 100,000	95%CI
Asia Pacific, High Income	22000	24.4	(11.8–46.2)	39000	45.1	(21.5–82.5)	62000	34.5	(19.2–57.2)
Asia Central	11000	27.7	(15.7–43.8)	20000	50.2	(29.2–81.0)	31000	38.7	(24.3–59.2)
Asia East	135000	20.1	(7.9–45.2)	247000	34.1	(14.3–71.0)	382000	27.3	(13.4–50.5)
Asia South	145000	18.6	(11.4–28.4)	270000	32.6	(19.7–48.7)	415000	25.8	(16.2–37.5)
Asia South East	56000	18.3	(10.4–29.8)	97000	32.0	(18.2–51.2)	153000	25.1	(15.8–37.7)
Australasia	8000	64.2	(41.5–93.7)	16000	122.5	(79.0–179.6)	24000	93.2	(61.7–134.2)
Caribbean	4000	18.1	(10.2–29.4)	7000	31.4	(17.6–49.5)	11000	24.7	(14.8–36.8)
Europe Central	14000	22.8	(13.6–35.8)	25000	43.9	(26.1–70.9)	39000	33.0	(20.5–50.1)
Europe Eastern	25000	22.9	(11.0–42.2)	43000	45.0	(22.3–78.6)	68000	33.1	(18.5–55.7)
Europe Western	64000	30.0	(18.4–44.6)	115000	56.5	(34.4–85.8)	179000	43.0	(27.0–62.3)
Latin America, Andean	4000	13.4	(6.3–23.0)	6000	23.0	(11.9–41.0)	10000	18.2	(10.2–29.8)
Latin America, Central	13000	11.2	(6.0–19.1)	22000	19.5	(10.5–32.7)	35000	15.3	(9.1–23.7)
Latin America, Southern	10000	31.0	(15.0–59.4)	17000	57.7	(28.6–106.7)	26000	44.1	(24.9–75.9)
Latin America, Tropical	17000	16.5	(6.5–34.5)	29000	29.4	(11.8–59.9)	46000	22.9	(11.4–41.0
North Africa/Middle East	40000	18.4	(11.4–28.6)	75000	33.0	(19.9–50.7)	115000	25.9	(16.2–37.7)
North America, High Income	98000	57.1	(37.0–82.1)	178000	106.4	(66.6–156.6)	276000	81.5	(53.6–116.6)
Oceania	1000	24.4	(11.1–47.0)	2000	41.4	(20.3–81.9)	3000	33.1	(17.4–57.6)
Sub-Saharan Africa Central	9000	17.7	(8.3–32.7)	14000	30.1	(14.5–57.0)	23000	23.9	(12.8–39.7)
Sub-Saharan Africa East	34000	18.8	(11.1–30.0)	58000	33.0	(19.9–51.7)	92000	25.9	(16.5–38.5)
Sub-Saharan Africa South	8000	23.6	(11.4–43.5)	15000	42.3	(20.9–78.8)	23000	32.8	(18.0–56.4)
Sub-Saharan Africa West	16000	9.6	(5.3–15.8)	27000	16.0	(9.2–27.0)	43000	12.8	(8.0–20.4)
**Global**	734000	21.5	(14.1–31.4)	1323000	38.1	(24.4–55.4)	2057000	29.9	(19.5–43.1)
