# Supplementary figures and images for: The Global Epidemiology and Contribution of Cannabis Use and Dependence to the Global Burden of Disease: Results from the GBD 2010 Study
Source: PLoS One. 2013 Oct 24;8(10):e76635. doi: 10.1371/journal.pone.0076635 (PMC3811989; doi:10.1371/journal.pone.0076635)

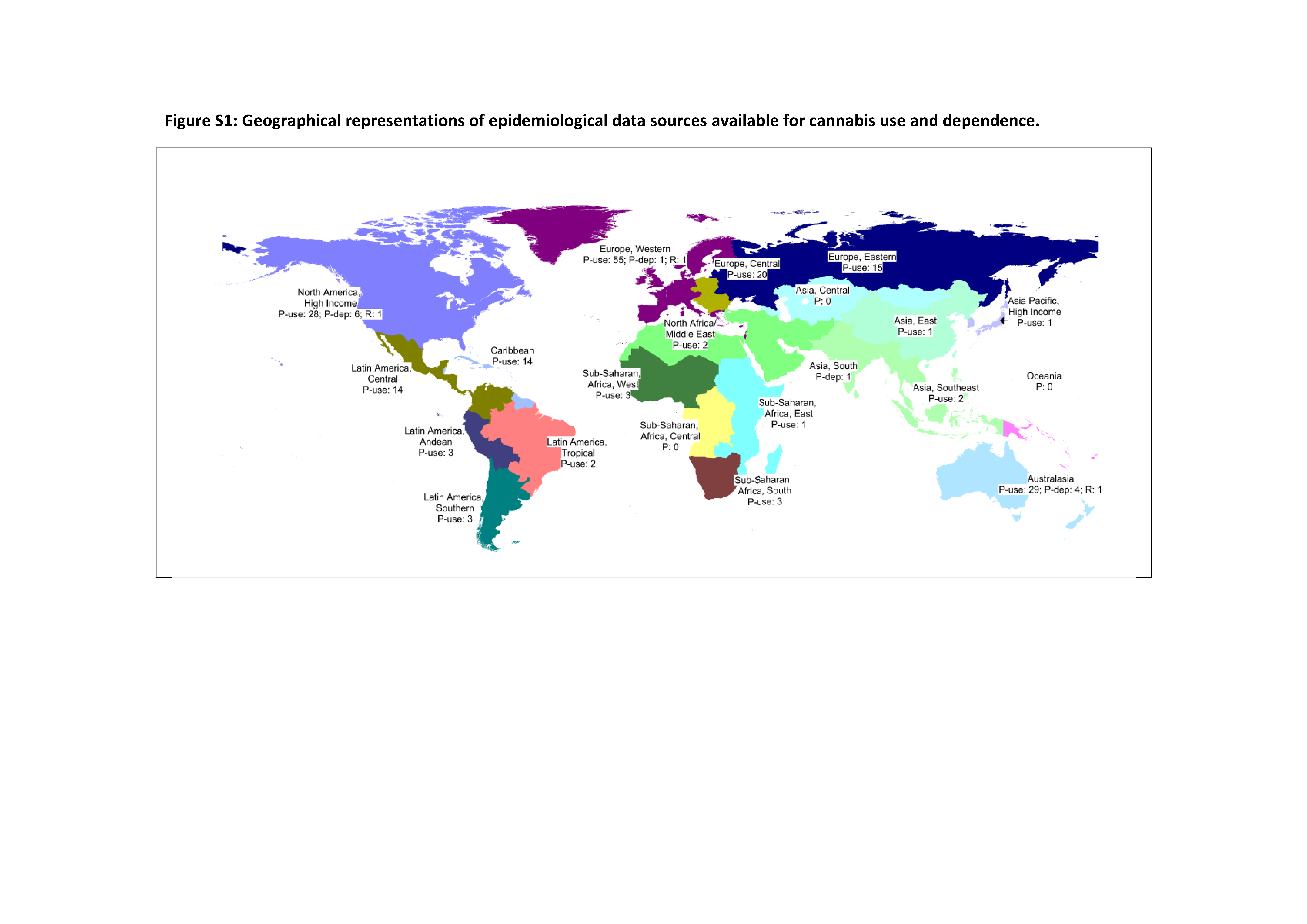

Supplement: Figure S1 — Geographical representations of epidemiological data sources available for cannabis use and dependence. Note. The world map shows the number of studies from each region included in the DisMod-MR modelling. Some studies reported estimates for more than one region; P-Use: Number of studies reporting on prevalence of cannabis use; P-dep: number of studies reporting on prevalence of cannabis dependence; R: number of studies reporting on remission from cannabis dependence; We also found 3 studies reporting on the incidence of cannabis dependence which have not been summarised here as they were not included in the DisMod-MR modelling. They were inconsistent to the data available for prevalence and remission. (TIF) [file pone.0076635.s001.tif]

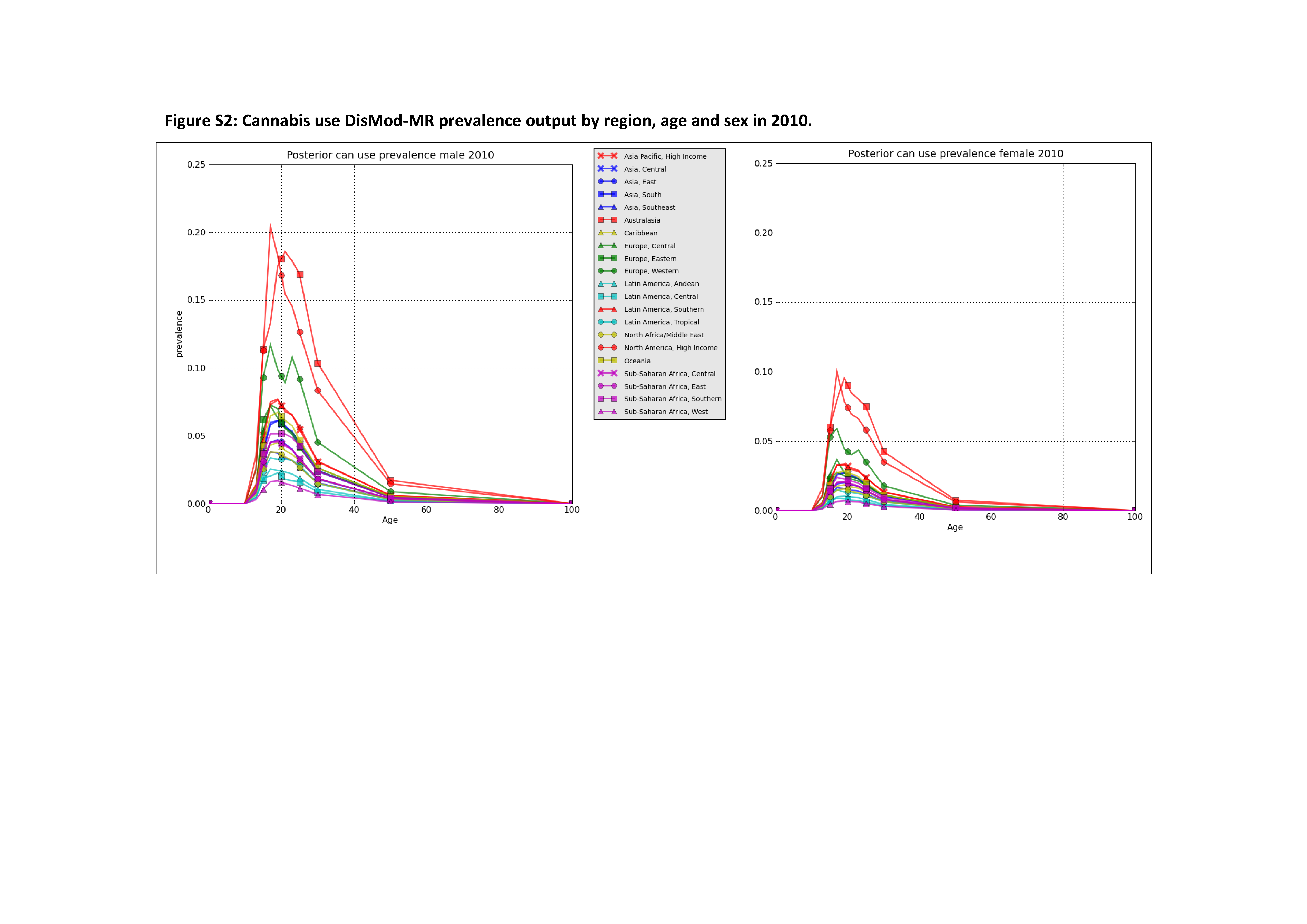

Supplement: Figure S2 — Cannabis use DisMod-MR prevalence output by region, age and sex in 2010. (TIF) [file pone.0076635.s002.tif]
