# Supplementary material for: The Global Epidemiology and Contribution of Cannabis Use and Dependence to the Global Burden of Disease: Results from the GBD 2010 Study
Source: PLoS One. 2013 Oct 24;8(10):e76635. doi: 10.1371/journal.pone.0076635 (PMC3811989; doi:10.1371/journal.pone.0076635)
Supplement: Table S1 — Summary of epidemiological data sources available for cannabis use and dependence. Note. References of the included data sources have been presented elsewhere[29-32]; *Studies may have reported estimates for the overall age group, age specific estimates falling within the overall age group, or both. (DOCX) [file pone.0076635.s003.docx]

**Table S1: Summary of epidemiological data sources available for cannabis use and dependence.**

| **GBD region** | **Number of studies** | **Number of data points** | **Disorder type** | **Parameter** | **Study period** | **Study age group (in years)*** |
| --- | --- | --- | --- | --- | --- | --- |
| Asia Pacific, High Income | 1 | 1 | Cannabis use | Prevalence | 2002-03 | 21 |
| Asia Central | - | - | - | - | - | - |
| Asia East | 1 | 1 | Cannabis use | Prevalence | 2002-03 | 25-29 |
| Asia South | 1 | 4 | Cannabis dependence | Prevalence | 1992-93, 2000 | 10-75 |
| Asia South East | - | 13 | Cannabis use | Prevalence | 2002, 2004-05 | 10-60 |
| Australasia | 34 | 208 | Cannabis use, dependence | Prevalence, Remission | 1992-93, 1995-98, 2000-01, 2003-05, 2007 | 12-99 |
| Caribbean | 14 | 67 | Cannabis use | Prevalence | 1997, 1999, 2002-03, 2005-07 | 10-75 |
| Europe Central | 20 | 114 | Cannabis use | Prevalence | 1995, 1999, 2002-06 | 15-64 |
| Europe Eastern | 14 | 60 | Cannabis use | Prevalence | 1995, 1999, 2001-04, 2006-07 | 15-69 |
| Europe Western | 57 | 509 | Cannabis use, dependence | Prevalence, Remission | 1998-07 | 10-75 |
| Latin America, Andean | 3 | 12 | Cannabis use | Prevalence | 2005 | 12-75 |
| Latin America, Central | 14 | 63 | Cannabis use | Prevalence | 1998, 2001-06 | 12-65 |
| Latin America, Southern | 3 | 19 | Cannabis use | Prevalence | 2003, 2005-06 | 12-65 |
| Latin America, Tropical | 2 | 24 | Cannabis use | Prevalence | 2003-05 | 12-54 |
| North Africa/Middle East | - | 9 | Cannabis use | Prevalence | 2002-03 | 15-59 |
| North America, High Income | 35 | 283 | Cannabis use, dependence | Prevalence, Remission | 1990-95, 1997-99, 2001-07 | 12-99 |
| Oceania | - | - | - | - | - | - |
| Sub-Saharan Africa Central | - | - | - | - | - | - |
| Sub-Saharan Africa East | 1 | 5 | Cannabis use | Prevalence | 1990, 2001 | 10-75 |
| Sub-Saharan Africa South | 3 | 22 | Cannabis use | Prevalence | 2002-03 | 18-79 |
| Sub-Saharan Africa West | 3 | 14 | Cannabis use | Prevalence | 2002-04, 2006-07 | 13-54 |
