# Supplementary material for: The Global Epidemiology and Contribution of Cannabis Use and Dependence to the Global Burden of Disease: Results from the GBD 2010 Study
Source: PLoS One. 2013 Oct 24;8(10):e76635. doi: 10.1371/journal.pone.0076635 (PMC3811989; doi:10.1371/journal.pone.0076635)
Supplement: Table S2 — Countries located in regions and super regions as defined in GBD 2010. (DOCX) [file pone.0076635.s004.docx]

**Table S2: Countries located in regions and super regions as defined in GBD 2010.**

| **GBD Super Region** | **GBD Region** | **Country** |
| --- | --- | --- |
| **OECD** | Asia Pacific, High Income | Brunei Darussalam, Japan, Republic of Korea (South Korea), Singapore |
|  | Australasia | Australia, New Zealand |
|  | Europe, Western | Akrotiri and Dhekelia, Aland Islands, Andorra, Austria, Belgium, Channel Islands, Cyprus, Denmark, Faeroe Islands, Finland, France, Germany, Gibraltar, Greece, Greenland, Holy See, Iceland, Ireland, Isle of Man, Israel, Italy, Liechtenstein, Luxembourg, Malta, Monaco, Netherlands, Norway, Portugal, San Marino, Spain, Sweden, Switzerland, United Kingdom |
|  | Latin America, Southern | Argentina, Chile, Falkland Islands (Malvinas), Uruguay |
|  | North America, High Income | Canada, Saint Pierre et Miquelon, United States of America |
| **Eastern Europe/Central Asia** | Asia, Central | Armenia, Azerbaijan, Georgia, Kazakhstan, Kyrgyzstan, Mongolia, Tajikistan, Turkmenistan, Uzbekistan |
|  | Europe, Central | Albania, Bosnia and Herzegovina, Bulgaria, Croatia, Czech Republic, Hungary, Kosovo, Montenegro, Poland, Romania, Serbia, Slovakia, Slovenia, The Former Yugoslav Republic of Macedonia |
|  | Europe, Eastern | Belarus, Estonia, Latvia, Lithuania, Republic of Moldova, Russian Federation, Ukraine |
| **Sub-Saharan Africa** | Sub-Saharan Africa, Central | Angola, Central African Republic, Congo, Democratic Republic of the Congo, Equatorial Guinea, Gabon |
|  | Sub-Saharan Africa, East | Burundi, Comoros, Djibouti, Eritrea, Ethiopia, Kenya, Madagascar, Malawi, Mayotte, Mozambique, Rwanda, Somalia, Sudan, Uganda, Tanzania (United Republic of), Zambia |
|  | Sub-Saharan Africa, Southern | Botswana, Lesotho, Namibia, South Africa, Swaziland, Zimbabwe |
|  | Sub-Saharan Africa, West | Benin, Burkina Faso, Cameroon, Cape Verde, Chad, Cote d’Ivoire, Gambia, Ghana, Guinea, Guinea-Bissau, Liberia, Mali, Mauritania, Niger, Nigeria, Saint Helena, Sao Tome and Principe, Senegal, Sierra Leone, Togo |
| **North Africa/Middle East** | North Africa/Middle East | Algeria, Bahrain, Egypt, Iran (Islamic Republic of), Iraq, Jordan, Kuwait, Lebanon, Libyan Arab Jamahiriya, Morocco, Occupied Palestinian Territory, Oman, Qatar, Saudi Arabia, Syrian Arab Republic, Tunisia, Turkey, United Arab Emirates, Western Sahara, Yemen |
| **South Asia** | Asia, South | Afghanistan, Bangladesh, Bhutan, India, Nepal, Pakistan |
|  | Asia, Southeast | Cambodia, Christmas Island, Cocos Islands, Indonesia, Lao People’s Democratic Republic, Malaysia, Maldives, Mauritius, Mayotte, Myanmar, Philippines, Reunion, Seychelles, Sri Lanka, Thailand, Timore-Leste, Viet Nam |
| **East Asia and Pacific** | Asia, East | China, Democratic People’s Republic of Korea (North Korea), Hong Kong, Taiwan |
|  | Oceania | American Samoa, Cook Islands, Fiji, French Polynesia, Guam, Kiribati, Marshall Islands, Micronesia (Federated States of), Nauru, New Caledonia, Niue, Northern Mariana Islands, Palau, Papua New Guinea, Pitcairn, Samoa, Solomon Islands, Tokelau, Tonga, Tuvalu, Vanuatu, Wallis and Futuna Islands |
| **Non-OECD Latin America/Caribbean** | Caribbean | Anguilla, Antigua and Barbuda, Aruba, Bahamas, Barbados, Belize, Bermuda, British Virgin Islands, Cayman Islands, Cuba, Dominica, Dominican Republic, French Guiana, Grenada, Guadeloupe, Guyana, Haiti, Jamaica, Martinique, Montserrat, Netherlands Antilles, Puerto Rico, Saint Kitts and Nevis, Saint Lucia, Saint Martin, Saint Vincent and the Grenadines, Suriname, Trinidad and Tobago, Turks and Caicos Islands, US Virgin Islands |
|  | Latin America, Andean | Bolivia, Ecuador, Peru |
|  | Latin America, Central | Colombia, Costa Rica, El Salvador, Guatemala, Honduras, Mexico, Nicaragua, Panama, Venezuela |
|  | Latin America, Tropical | Brazil, Paraguay |
