# Supplementary material for: The Global Epidemiology and Contribution of Cannabis Use and Dependence to the Global Burden of Disease: Results from the GBD 2010 Study
Source: PLoS One. 2013 Oct 24;8(10):e76635. doi: 10.1371/journal.pone.0076635 (PMC3811989; doi:10.1371/journal.pone.0076635)
Supplement: Table S3 — Estimated prevalence and number of cases of cannabis dependence in 1990, by sex and GBD region. (DOCX) [file pone.0076635.s005.docx]

**Table S3: Estimated prevalence and number of cases of cannabis dependence in 1990, by sex and GBD region.**

|  | Females | | | Males | | | Total | | |
| --- | --- | --- | --- | --- | --- | --- | --- | --- | --- |
|  | N | % | 95%CI | N | % | 95%CI | N | % | 95%CI |
| Asia Pacific, High Income | 170000 | 0.2% | (0.1-0.3) | 294000 | 0.3% | (0.2-0.6) | 464000 | 0.3% | (0.2-0.4) |
| Asia Central | 56000 | 0.16% | (0.1-0.2) | 96000 | 0.3% | (0.2-0.4) | 151000 | 0.2% | (0.17-0.3) |
| Asia East | 821000 | 0.12% | (0.1-0.2) | 1485000 | 0.2% | (0.1-0.4) | 2306000 | 0.2% | (0.1-0.3) |
| Asia South | 600000 | 0.1% | (0.09-0.14) | 1121000 | 0.18% | (0.1-0.2) | 1720000 | 0.14% | (0.1-0.2) |
| Asia South East | 270000 | 0.1% | (0.07-0.2) | 465000 | 0.2% | (0.1-0.3) | 734000 | 0.14% | (0.1-0.2) |
| Australasia | 49000 | 0.5% | (0.4-0.36) | 90000 | 0.9% | (0.7-1.0) | 139000 | 0.7% | (0.6-0.8) |
| Caribbean | 23000 | 0.12% | (0.1-0.2) | 38000 | 0.2% | (0.1-0.3) | 60000 | 0.16% | (0.1-0.2) |
| Europe Central | 92000 | 0.17% | (0.1-0.2) | 164000 | 0.3% | (0.2-0.4) | 255000 | 0.2% | (0.18-0.3) |
| Europe Eastern | 162000 | 0.2% | (0.1-0.3) | 272000 | 0.3% | (0.2-0.4) | 435000 | 0.2% | (0.1-0.3) |
| Europe Western | 453000 | 0.25% | (0.2-0.3) | 811000 | 0.4% | (0.3-0.6) | 1263000 | 0.3% | (0.28-0.4) |
| Latin America, Andean | 16000 | 0.08% | (0.04-0.1) | 28000 | 0.14% | (0.1-0.2) | 44000 | 0.1% | (0.07-0.2) |
| Latin America, Central | 60000 | 0.07% | (0.04-0.1) | 100000 | 0.1% | (0.07-0.2) | 161000 | 0.1% | (0.07-0.12) |
| Latin America, Southern | 50000 | 0.2% | (0.1-0.4) | 83000 | 0.3% | (0.2-0.6) | 132000 | 0.3% | (0.2-0.4) |
| Latin America, Tropical | 82000 | 0.1% | (0.05-0.2) | 140000 | 0.2% | (0.1-0.3) | 222000 | 0.1% | (0.07-0.2) |
| North Africa/Middle East | 156000 | 0.1% | (0.08-0.14) | 272000 | 0.18% | (0.1-0.2) | 427000 | 0.14% | (0.1-0.2) |
| North America, High Income | 567000 | 0.4% | (0.3-0.5) | 997000 | 0.7% | (0.6-0.9) | 1564000 | 0.6% | (0.5-0.7) |
| Oceania | 5000 | 0.2% | (0.1-0.3) | 9000 | 0.3% | (0.1-0.4) | 14000 | 0.2% | (0.1-0.3) |
| Sub-Saharan Africa Central | 29000 | 0.1% | (0.07-0.2) | 50000 | 0.2% | (0.1-0.3) | 78000 | 0.15% | (0.1-0.2) |
| Sub-Saharan Africa East | 122000 | 0.1% | (0.08-0.2) | 205000 | 0.2% | (0.15-0.3) | 327000 | 0.16% | (0.1-0.2) |
| Sub-Saharan Africa South | 38000 | 0.1% | (0.08-0.2) | 64000 | 0.2% | (0.1-0.4) | 101000 | 0.2% | (0.1-0.3) |
| Sub-Saharan Africa West | 56000 | 0.06% | (0.04-0.1) | 97000 | 0.1% | (0.07-0.2) | 152000 | 0.08% | (0.06-0.1) |
| Global | 3874000 | 0.14% | (0.1-0.2) | 6879000 | 0.2% | (0.21-0.3) | 10752000 | 0.2% | (0.17-0.22) |
