# Supplementary material for: The Global Epidemiology and Contribution of Cannabis Use and Dependence to the Global Burden of Disease: Results from the GBD 2010 Study
Source: PLoS One. 2013 Oct 24;8(10):e76635. doi: 10.1371/journal.pone.0076635 (PMC3811989; doi:10.1371/journal.pone.0076635)
Supplement: Table S4 — Country-level estimates of cannabis dependence and DALYs per 100,000 due to cannabis dependence, sex and age-standardised, 2010. (DOCX) [file pone.0076635.s006.docx]

**Table S4: Country-level estimates of cannabis dependence and DALYs per 100,000 due to cannabis dependence, sex and age-standardised, 2010.**

| Region | **Country** | **Cannabis dependence** | | | **Cannabis dependence DALYs** | | |
| --- | --- | --- | --- | --- | --- | --- | --- |
|  |  | **Lower %** | **%** | **Upper %** | **Lower** | **Rate per 100,000** | **Upper** |
| Asia Pacific, High Income | Brunei | 0.16% | 0.28% | 0.47% | 21.0 | 43.6 | 79.7 |
| Asia Pacific, High Income | Japan | 0.15% | 0.27% | 0.45% | 21.5 | 43.5 | 81.1 |
| Asia Pacific, High Income | Singapore | 0.16% | 0.27% | 0.46% | 19.8 | 43.3 | 80.3 |
| Asia Pacific, High Income | South Korea | 0.16% | 0.28% | 0.46% | 21.2 | 43.8 | 80.6 |
| Asia, Central | Armenia | 0.12% | 0.22% | 0.37% | 16.2 | 34.0 | 60.9 |
| Asia, Central | Azerbaijan | 0.13% | 0.22% | 0.37% | 16.7 | 34.1 | 62.2 |
| Asia, Central | Georgia | 0.12% | 0.22% | 0.36% | 16.4 | 34.0 | 61.4 |
| Asia, Central | Kazakhstan | 0.12% | 0.22% | 0.37% | 17.0 | 34.1 | 64.2 |
| Asia, Central | Kyrgyzstan | 0.12% | 0.22% | 0.37% | 16.5 | 33.5 | 58.5 |
| Asia, Central | Mongolia | 0.12% | 0.22% | 0.36% | 16.3 | 33.8 | 62.5 |
| Asia, Central | Tajikistan | 0.12% | 0.21% | 0.35% | 16.3 | 33.2 | 61.1 |
| Asia, Central | Turkmenistan | 0.12% | 0.22% | 0.36% | 16.4 | 33.7 | 62.3 |
| Asia, Central | Uzbekistan | 0.12% | 0.22% | 0.36% | 17.0 | 34.2 | 59.3 |
| Asia, East | China | 0.09% | 0.17% | 0.28% | 12.8 | 26.5 | 49.5 |
| Asia, East | North Korea | 0.09% | 0.17% | 0.29% | 12.4 | 26.3 | 48.4 |
| Asia, East | Taiwan | 0.09% | 0.16% | 0.27% | 12.8 | 26.1 | 47.8 |
| Asia, South | Afghanistan | 0.08% | 0.14% | 0.25% | 10.5 | 22.0 | 42.8 |
| Asia, South | Bangladesh | 0.09% | 0.16% | 0.27% | 11.3 | 24.6 | 44.9 |
| Asia, South | Bhutan | 0.09% | 0.16% | 0.27% | 12.1 | 25.2 | 47.0 |
| Asia, South | India | 0.13% | 0.15% | 0.18% | 14.7 | 23.5 | 34.1 |
| Asia, South | Nepal | 0.09% | 0.16% | 0.27% | 11.7 | 24.3 | 45.9 |
| Asia, South | Pakistan | 0.09% | 0.16% | 0.26% | 11.8 | 25.1 | 46.5 |
| Asia, Southeast | Cambodia | 0.08% | 0.15% | 0.25% | 11.4 | 23.2 | 42.2 |
| Asia, Southeast | Indonesia | 0.08% | 0.15% | 0.24% | 11.7 | 23.1 | 40.2 |
| Asia, Southeast | Laos | 0.08% | 0.15% | 0.26% | 11.2 | 23.4 | 43.7 |
| Asia, Southeast | Malaysia | 0.08% | 0.15% | 0.24% | 11.3 | 23.2 | 43.6 |
| Asia, Southeast | Maldives | 0.08% | 0.15% | 0.25% | 10.7 | 23.6 | 43.0 |
| Asia, Southeast | Mauritius | 0.08% | 0.15% | 0.24% | 11.5 | 23.5 | 43.5 |
| Asia, Southeast | Myanmar | 0.08% | 0.15% | 0.24% | 10.7 | 23.1 | 39.9 |
| Asia, Southeast | Philippines | 0.08% | 0.15% | 0.24% | 11.0 | 23.2 | 41.7 |
| Asia, Southeast | Seychelles | 0.08% | 0.15% | 0.25% | 11.2 | 23.2 | 41.4 |
| Asia, Southeast | Sri Lanka | 0.08% | 0.15% | 0.25% | 11.1 | 23.1 | 44.0 |
| Asia, Southeast | Thailand | 0.08% | 0.15% | 0.25% | 11.1 | 23.1 | 42.3 |
| Asia, Southeast | Timor-Leste | 0.08% | 0.15% | 0.25% | 11.1 | 23.2 | 43.6 |
| Asia, Southeast | Vietnam | 0.08% | 0.15% | 0.24% | 11.5 | 23.2 | 41.3 |
| Australasia | Australia | 0.57% | 0.67% | 0.78% | 69.0 | 104.9 | 152.6 |
| Australasia | New Zealand | 0.62% | 0.73% | 0.86% | 75.9 | 115.2 | 166.4 |
| Caribbean | Antigua and Barbuda | 0.09% | 0.15% | 0.26% | 12.1 | 24.0 | 44.7 |
| Caribbean | Barbados | 0.09% | 0.16% | 0.27% | 11.6 | 24.8 | 46.1 |
| Caribbean | Belize | 0.09% | 0.16% | 0.27% | 12.6 | 24.6 | 45.5 |
| Caribbean | Cuba | 0.09% | 0.16% | 0.26% | 11.2 | 24.3 | 43.7 |
| Caribbean | Dominica | 0.08% | 0.16% | 0.25% | 11.5 | 24.2 | 44.4 |
| Caribbean | Dominican Republic | 0.09% | 0.16% | 0.26% | 12.3 | 24.6 | 45.5 |
| Caribbean | Grenada | 0.09% | 0.16% | 0.27% | 12.5 | 24.7 | 44.1 |
| Caribbean | Guyana | 0.09% | 0.15% | 0.25% | 12.6 | 23.9 | 42.9 |
| Caribbean | Haiti | 0.09% | 0.16% | 0.26% | 11.4 | 23.7 | 41.8 |
| Caribbean | Jamaica | 0.09% | 0.16% | 0.26% | 12.0 | 24.4 | 43.0 |
| Caribbean | Saint Lucia | 0.09% | 0.16% | 0.27% | 12.0 | 24.1 | 45.2 |
| Caribbean | St. Vincent & the Grenadines | 0.09% | 0.16% | 0.26% | 12.6 | 24.8 | 44.3 |
| Caribbean | Suriname | 0.09% | 0.16% | 0.26% | 12.6 | 24.5 | 44.1 |
| Caribbean | The Bahamas | 0.09% | 0.16% | 0.26% | 12.4 | 24.9 | 45.1 |
| Caribbean | Trinidad and Tobago | 0.09% | 0.16% | 0.27% | 11.7 | 24.6 | 46.1 |
| Europe, Central | Albania | 0.13% | 0.22% | 0.37% | 17.2 | 34.8 | 63.5 |
| Europe, Central | Bosnia and Herzegovina | 0.13% | 0.22% | 0.37% | 17.3 | 35.1 | 65.8 |
| Europe, Central | Bulgaria | 0.13% | 0.22% | 0.37% | 17.7 | 35.4 | 66.3 |
| Europe, Central | Croatia | 0.12% | 0.22% | 0.38% | 17.2 | 35.1 | 65.0 |
| Europe, Central | Czech Republic | 0.13% | 0.23% | 0.39% | 18.0 | 35.6 | 66.2 |
| Europe, Central | Hungary | 0.12% | 0.22% | 0.38% | 16.0 | 34.6 | 64.0 |
| Europe, Central | Macedonia | 0.12% | 0.23% | 0.38% | 16.2 | 35.7 | 65.8 |
| Europe, Central | Montenegro | 0.13% | 0.23% | 0.39% | 16.9 | 35.4 | 66.3 |
| Europe, Central | Poland | 0.13% | 0.23% | 0.38% | 17.1 | 35.8 | 69.5 |
| Europe, Central | Romania | 0.13% | 0.23% | 0.38% | 17.9 | 35.7 | 65.8 |
| Europe, Central | Serbia | 0.13% | 0.23% | 0.39% | 16.8 | 35.7 | 66.9 |
| Europe, Central | Slovakia | 0.13% | 0.23% | 0.38% | 17.6 | 36.0 | 67.7 |
| Europe, Central | Slovenia | 0.13% | 0.23% | 0.38% | 18.4 | 35.8 | 67.9 |
|  |  |  |  |  |  |  |  |
| Europe, Eastern | Belarus | 0.12% | 0.22% | 0.37% | 17.2 | 34.1 | 62.1 |
| Europe, Eastern | Estonia | 0.12% | 0.22% | 0.35% | 15.9 | 34.1 | 61.1 |
| Europe, Eastern | Latvia | 0.12% | 0.22% | 0.37% | 16.6 | 34.4 | 60.2 |
| Europe, Eastern | Lithuania | 0.12% | 0.22% | 0.37% | 16.7 | 34.7 | 63.7 |
| Europe, Eastern | Moldova | 0.12% | 0.22% | 0.37% | 16.1 | 33.9 | 62.8 |
| Europe, Eastern | Russia | 0.13% | 0.22% | 0.37% | 16.3 | 34.4 | 65.0 |
| Europe, Eastern | Ukraine | 0.13% | 0.22% | 0.35% | 17.2 | 33.9 | 58.5 |
| Europe, Western | Andorra | 0.19% | 0.34% | 0.58% | 25.5 | 53.0 | 96.7 |
| Europe, Western | Austria | 0.19% | 0.35% | 0.58% | 26.1 | 54.3 | 99.4 |
| Europe, Western | Belgium | 0.19% | 0.34% | 0.57% | 25.4 | 53.9 | 94.0 |
| Europe, Western | Cyprus | 0.19% | 0.34% | 0.58% | 27.5 | 53.8 | 96.0 |
| Europe, Western | Denmark | 0.20% | 0.34% | 0.57% | 26.4 | 54.0 | 95.3 |
| Europe, Western | Finland | 0.20% | 0.34% | 0.58% | 26.0 | 53.9 | 98.0 |
| Europe, Western | France | 0.20% | 0.35% | 0.59% | 26.5 | 54.3 | 100.7 |
| Europe, Western | Germany | 0.27% | 0.32% | 0.39% | 32.8 | 51.0 | 73.9 |
| Europe, Western | Greece | 0.19% | 0.34% | 0.58% | 25.9 | 54.2 | 95.4 |
| Europe, Western | Iceland | 0.20% | 0.35% | 0.58% | 26.5 | 54.3 | 102.2 |
| Europe, Western | Ireland | 0.19% | 0.34% | 0.56% | 27.4 | 53.4 | 99.3 |
| Europe, Western | Israel | 0.19% | 0.34% | 0.58% | 25.8 | 53.5 | 100.0 |
| Europe, Western | Italy | 0.19% | 0.33% | 0.58% | 25.6 | 52.4 | 94.3 |
| Europe, Western | Luxembourg | 0.19% | 0.34% | 0.55% | 25.5 | 53.5 | 94.5 |
| Europe, Western | Malta | 0.19% | 0.35% | 0.59% | 26.6 | 54.3 | 99.6 |
| Europe, Western | Netherlands | 0.19% | 0.34% | 0.57% | 26.2 | 53.5 | 94.2 |
| Europe, Western | Norway | 0.20% | 0.34% | 0.57% | 27.3 | 53.7 | 93.9 |
| Europe, Western | Portugal | 0.20% | 0.35% | 0.58% | 26.4 | 54.2 | 100.7 |
| Europe, Western | Spain | 0.19% | 0.35% | 0.57% | 27.1 | 54.8 | 99.9 |
| Europe, Western | Sweden | 0.19% | 0.34% | 0.56% | 24.8 | 53.7 | 95.5 |
| Europe, Western | Switzerland | 0.19% | 0.34% | 0.59% | 26.0 | 54.1 | 102.2 |
| Europe, Western | United Kingdom | 0.19% | 0.34% | 0.55% | 26.3 | 53.4 | 98.1 |
| Latin America, Andean | Bolivia | 0.06% | 0.11% | 0.18% | 8.3 | 16.9 | 31.8 |
| Latin America, Andean | Ecuador | 0.06% | 0.11% | 0.18% | 8.5 | 17.2 | 31.0 |
| Latin America, Andean | Peru | 0.06% | 0.11% | 0.18% | 8.1 | 17.0 | 30.8 |
| Latin America, Central | Colombia | 0.05% | 0.09% | 0.15% | 7.1 | 14.3 | 26.6 |
| Latin America, Central | Costa Rica | 0.05% | 0.09% | 0.15% | 6.7 | 14.5 | 26.2 |
| Latin America, Central | El Salvador | 0.05% | 0.09% | 0.14% | 6.9 | 13.7 | 24.8 |
| Latin America, Central | Guatemala | 0.05% | 0.09% | 0.15% | 6.7 | 14.2 | 27.2 |
| Latin America, Central | Honduras | 0.05% | 0.09% | 0.15% | 7.1 | 14.2 | 26.4 |
| Latin America, Central | Mexico | 0.05% | 0.09% | 0.17% | 7.0 | 14.7 | 26.8 |
| Latin America, Central | Nicaragua | 0.05% | 0.09% | 0.15% | 6.7 | 14.1 | 25.4 |
| Latin America, Central | Panama | 0.05% | 0.09% | 0.15% | 7.1 | 14.5 | 26.2 |
| Latin America, Central | Venezuela | 0.05% | 0.09% | 0.15% | 6.6 | 14.4 | 26.5 |
| Latin America, Southern | Argentina | 0.15% | 0.28% | 0.48% | 21.9 | 44.5 | 80.9 |
| Latin America, Southern | Chile | 0.16% | 0.28% | 0.48% | 22.0 | 44.7 | 81.2 |
| Latin America, Southern | Uruguay | 0.16% | 0.28% | 0.46% | 21.8 | 44.5 | 79.0 |
| Latin America, Tropical | Brazil | 0.07% | 0.14% | 0.23% | 10.5 | 21.6 | 39.1 |
| Latin America, Tropical | Paraguay | 0.08% | 0.14% | 0.22% | 10.5 | 21.3 | 39.0 |
| North Africa / Middle East | Algeria | 0.08% | 0.15% | 0.25% | 10.6 | 22.8 | 42.0 |
| North Africa / Middle East | Bahrain | 0.08% | 0.15% | 0.25% | 11.4 | 23.4 | 42.7 |
| North Africa / Middle East | Egypt | 0.08% | 0.14% | 0.23% | 10.5 | 22.3 | 39.8 |
| North Africa / Middle East | Iran | 0.08% | 0.15% | 0.25% | 10.2 | 22.7 | 40.9 |
| North Africa / Middle East | Iraq | 0.08% | 0.14% | 0.24% | 11.0 | 22.6 | 42.6 |
| North Africa / Middle East | Jordan | 0.08% | 0.15% | 0.24% | 11.0 | 22.7 | 42.8 |
| North Africa / Middle East | Kuwait | 0.09% | 0.15% | 0.26% | 11.2 | 23.7 | 43.0 |
| North Africa / Middle East | Lebanon | 0.08% | 0.14% | 0.24% | 10.5 | 22.5 | 40.9 |
| North Africa / Middle East | Libya | 0.08% | 0.14% | 0.24% | 10.4 | 22.4 | 42.3 |
| North Africa / Middle East | Morocco | 0.08% | 0.14% | 0.23% | 10.7 | 21.9 | 39.8 |
| North Africa / Middle East | Oman | 0.08% | 0.15% | 0.26% | 11.3 | 23.5 | 44.6 |
| North Africa / Middle East | Palestine | 0.08% | 0.15% | 0.25% | 10.9 | 22.7 | 41.4 |
| North Africa / Middle East | Qatar | 0.08% | 0.16% | 0.30% | 11.2 | 25.6 | 49.4 |
| North Africa / Middle East | Saudi Arabia | 0.08% | 0.15% | 0.25% | 11.1 | 22.8 | 40.7 |
| North Africa / Middle East | Syria | 0.08% | 0.14% | 0.25% | 10.5 | 22.5 | 44.3 |
| North Africa / Middle East | Tunisia | 0.08% | 0.14% | 0.24% | 11.3 | 22.7 | 40.8 |
| North Africa / Middle East | Turkey | 0.08% | 0.14% | 0.24% | 11.4 | 22.4 | 41.4 |
| North Africa / Middle East | United Arab Emirates | 0.08% | 0.16% | 0.27% | 11.6 | 24.6 | 47.8 |
| North Africa / Middle East | Yemen | 0.08% | 0.14% | 0.23% | 10.7 | 22.1 | 41.2 |
|  |  |  |  |  |  |  |  |
| North America, High Income | Canada | 0.50% | 0.60% | 0.71% | 61.2 | 94.9 | 135.1 |
| North America, High Income | United States | 0.52% | 0.60% | 0.69% | 61.6 | 94.0 | 135.3 |
| Oceania | Fed. States of Micronesia | 0.11% | 0.21% | 0.34% | 15.7 | 32.2 | 61.3 |
| Oceania | Fiji | 0.11% | 0.21% | 0.36% | 15.4 | 32.0 | 62.9 |
| Oceania | Kiribati | 0.11% | 0.21% | 0.35% | 15.4 | 31.9 | 55.7 |
| Oceania | Marshall Islands | 0.12% | 0.21% | 0.34% | 15.2 | 32.1 | 57.8 |
| Oceania | Papua New Guinea | 0.11% | 0.20% | 0.35% | 15.1 | 31.8 | 61.1 |
| Oceania | Samoa | 0.11% | 0.21% | 0.35% | 15.5 | 32.4 | 57.8 |
| Oceania | Solomon Islands | 0.11% | 0.20% | 0.35% | 14.7 | 31.8 | 60.9 |
| Oceania | Tonga | 0.11% | 0.21% | 0.35% | 15.2 | 32.4 | 62.6 |
| Oceania | Vanuatu | 0.11% | 0.21% | 0.35% | 15.4 | 32.1 | 55.8 |
| Sub-Saharan Africa, Central | Angola | 0.09% | 0.16% | 0.26% | 12.0 | 24.6 | 45.7 |
| Sub-Saharan Africa, Central | Central African Republic | 0.09% | 0.16% | 0.27% | 11.3 | 24.6 | 43.0 |
| Sub-Saharan Africa, Central | Congo | 0.09% | 0.16% | 0.27% | 11.7 | 24.5 | 46.9 |
| Sub-Saharan Africa, Central | Dem. Republic of the Congo | 0.09% | 0.16% | 0.25% | 11.4 | 24.2 | 44.5 |
| Sub-Saharan Africa, Central | Equatorial Guinea | 0.09% | 0.16% | 0.27% | 12.1 | 24.7 | 45.5 |
| Sub-Saharan Africa, Central | Gabon | 0.09% | 0.16% | 0.28% | 11.6 | 24.6 | 47.4 |
| Sub-Saharan Africa, East | Burundi | 0.09% | 0.16% | 0.28% | 12.8 | 25.7 | 49.6 |
| Sub-Saharan Africa, East | Comoros | 0.09% | 0.16% | 0.27% | 11.7 | 25.5 | 49.4 |
| Sub-Saharan Africa, East | Djibouti | 0.09% | 0.16% | 0.28% | 12.1 | 25.5 | 46.7 |
| Sub-Saharan Africa, East | Eritrea | 0.09% | 0.17% | 0.28% | 12.0 | 25.7 | 48.6 |
| Sub-Saharan Africa, East | Ethiopia | 0.09% | 0.16% | 0.28% | 11.9 | 25.5 | 48.1 |
| Sub-Saharan Africa, East | Kenya | 0.09% | 0.16% | 0.29% | 12.5 | 25.4 | 45.7 |
| Sub-Saharan Africa, East | Madagascar | 0.09% | 0.16% | 0.28% | 12.4 | 24.9 | 44.0 |
| Sub-Saharan Africa, East | Malawi | 0.09% | 0.16% | 0.27% | 12.5 | 25.3 | 46.6 |
| Sub-Saharan Africa, East | Mozambique | 0.09% | 0.16% | 0.26% | 12.0 | 24.7 | 43.9 |
| Sub-Saharan Africa, East | Rwanda | 0.09% | 0.16% | 0.28% | 13.2 | 25.5 | 47.8 |
| Sub-Saharan Africa, East | Somalia | 0.09% | 0.16% | 0.27% | 12.4 | 25.1 | 46.9 |
| Sub-Saharan Africa, East | Sudan | 0.09% | 0.16% | 0.27% | 12.3 | 25.3 | 47.8 |
| Sub-Saharan Africa, East | Tanzania | 0.09% | 0.16% | 0.26% | 12.0 | 25.1 | 44.9 |
| Sub-Saharan Africa, East | Uganda | 0.09% | 0.16% | 0.27% | 12.3 | 25.4 | 46.1 |
| Sub-Saharan Africa, East | Zambia | 0.09% | 0.16% | 0.27% | 12.5 | 25.4 | 46.4 |
| Sub-Saharan Africa, Southern | Botswana | 0.10% | 0.19% | 0.30% | 13.6 | 28.9 | 54.1 |
| Sub-Saharan Africa, Southern | Lesotho | 0.10% | 0.19% | 0.32% | 13.3 | 28.8 | 54.9 |
| Sub-Saharan Africa, Southern | Namibia | 0.10% | 0.18% | 0.32% | 13.7 | 28.6 | 53.5 |
| Sub-Saharan Africa, Southern | South Africa | 0.10% | 0.18% | 0.31% | 13.8 | 28.7 | 55.0 |
| Sub-Saharan Africa, Southern | Swaziland | 0.10% | 0.18% | 0.30% | 13.6 | 28.3 | 51.3 |
| Sub-Saharan Africa, Southern | Zimbabwe | 0.11% | 0.19% | 0.30% | 14.0 | 28.8 | 52.0 |
| Sub-Saharan Africa, West | Benin | 0.05% | 0.08% | 0.14% | 6.0 | 12.5 | 22.8 |
| Sub-Saharan Africa, West | Burkina Faso | 0.05% | 0.08% | 0.13% | 6.0 | 12.6 | 22.8 |
| Sub-Saharan Africa, West | Cameroon | 0.04% | 0.08% | 0.14% | 6.0 | 12.7 | 23.7 |
| Sub-Saharan Africa, West | Cape Verde | 0.05% | 0.08% | 0.14% | 6.4 | 13.0 | 23.9 |
| Sub-Saharan Africa, West | Chad | 0.05% | 0.08% | 0.13% | 6.1 | 12.5 | 22.0 |
| Sub-Saharan Africa, West | Côte d’Ivoire | 0.04% | 0.08% | 0.14% | 6.1 | 12.8 | 23.6 |
| Sub-Saharan Africa, West | Ghana | 0.04% | 0.08% | 0.14% | 5.8 | 12.5 | 22.7 |
| Sub-Saharan Africa, West | Guinea | 0.04% | 0.08% | 0.14% | 6.1 | 12.7 | 23.8 |
| Sub-Saharan Africa, West | Guinea-Bissau | 0.04% | 0.08% | 0.14% | 6.1 | 12.5 | 22.8 |
| Sub-Saharan Africa, West | Liberia | 0.05% | 0.08% | 0.14% | 6.0 | 12.6 | 23.6 |
| Sub-Saharan Africa, West | Mali | 0.05% | 0.08% | 0.13% | 6.2 | 12.5 | 23.4 |
| Sub-Saharan Africa, West | Mauritania | 0.05% | 0.08% | 0.14% | 6.2 | 12.6 | 22.4 |
| Sub-Saharan Africa, West | Niger | 0.04% | 0.08% | 0.13% | 6.0 | 12.6 | 23.2 |
| Sub-Saharan Africa, West | Nigeria | 0.04% | 0.08% | 0.14% | 6.0 | 12.6 | 23.4 |
| Sub-Saharan Africa, West | São Tomé and Príncipe | 0.05% | 0.08% | 0.13% | 5.9 | 12.4 | 22.2 |
| Sub-Saharan Africa, West | Senegal | 0.04% | 0.08% | 0.13% | 6.0 | 12.4 | 23.6 |
| Sub-Saharan Africa, West | Sierra Leone | 0.04% | 0.08% | 0.13% | 5.7 | 12.2 | 21.6 |
| Sub-Saharan Africa, West | The Gambia | 0.04% | 0.08% | 0.14% | 5.8 | 12.4 | 23.2 |
| Sub-Saharan Africa, West | Togo | 0.05% | 0.08% | 0.14% | 5.9 | 12.4 | 23.2 |
