# Supplementary material for: The Global Epidemiology and Contribution of Cannabis Use and Dependence to the Global Burden of Disease: Results from the GBD 2010 Study
Source: PLoS One. 2013 Oct 24;8(10):e76635. doi: 10.1371/journal.pone.0076635 (PMC3811989; doi:10.1371/journal.pone.0076635)
Supplement: Text S1 — Additional information on the estimation of burden for cannabis dependence in GBD 2010. (DOCX) [file pone.0076635.s007.docx]

**Text S1: Additional information on the estimation of burden for cannabis dependence in GBD 2010.**

**Disability weights**

Full details of the disability weight generation process have been described in detail elsewhere[^5^](#_ENREF_5). In brief, household surveys were completed by community samples in five countries (Bangladesh, Indonesia, Peru, the United Republic of Tanzania and the United States of America) and by respondents to an open-access internet survey.

Standardised survey instruments were developed to elicit comparative assessments on the health consequences associated with the 220 unique health states in GBD 2010, including cannabis dependence. The basis for all comparisons were brief lay descriptions that highlighted the major functional consequences and symptoms associated with each health state using simple, nonclinical vocabulary. The description for cannabis dependence is presented in the box below.

This person uses marijuana daily and has difficulty controlling the habit. The person sometimes has mood swings, anxiety and hallucinations, and has some difficulty in daily activities.

Assessments of nonfatal health outcomes were elicited using a paired comparison question, in which respondents were presented with two descriptions of hypothetical people, each living in a particular health state, and then asked which person they regarded as being healthier.

Probit regression analyses were run on a pooled dataset that contained 379,492 paired comparison responses, and on datasets for each individual survey. Comparing results from regression analyses for each individual survey with pooled data, there was a high degree of consistency between sites. Pearson’s correlation coefficients between individual survey results and the results from the pooled analyses were 0·9 or higher in all cases except Bangladesh[^5^](#_ENREF_5). Comparing the web survey, which had the most highly educated respondents, with the survey in Tanzania, which had respondents with the lowest average educational levels, a correlation of 0·89 was found. Comparisons based on Lin’s concordance correlation coefficient were virtually identical to the Pearson’s results in most cases; the largest discrepancy between the two measures appeared in Bangladesh, where Lin’s coefficient was 0·72[^5^](#_ENREF_5). Visual depictions of these findings are presented in detail in the original paper documenting this work[^5^](#_ENREF_5).

**Severity proportions**

GBD 2010 also used survey data to adjust each disability weight for the severity of disorder[^5-8^](#_ENREF_5). For cannabis dependence, these data came from the USA National Epidemiologic Survey on Alcohol and Related Conditions (NESARC)[^9^](#_ENREF_9), which used the Short Form 12-Item Survey (SF-12)[^10^](#_ENREF_10) to capture disability. First, a sample of 2202 participants from the *GBD Health Measurement Survey*[*^5^*](#_ENREF_5) were asked to fill in the SF-12 to match the lay descriptions of 62 health states of diverse severity.

These data were used to derive a mathematical relationship between SF-12 scores from NESARC and a GBD 2010 disability weight[^11^](#_ENREF_11). NESARC participants were each allocated their corresponding GBD 2010 disability weight. Secondly, regression methods were used to calculate average disability weights for each NESARC condition, while adjusting for any other co-morbid condition. As with other diseases included in GBD 2010, a proportion of NESARC participants with cannabis dependence were rated as having no disability (i.e. a disability weight of 0) at the time of the survey after the disability attributable to comorbid disorders had been portioned out. The unadjusted disability weight for cannabis dependence (from the *GBD Health Measurement Survey*) was 0·329 (0·223–0·455)[^5^](#_ENREF_5). After adjusting for an estimated 51% (47%-54%) of cases who had no disability and 49% (46-53%) of cases with disability (after accounting for comorbidity), the average disability weight was 0.162 (0.109 to 0.224). Further details are presented elsewhere[^6-8^](#_ENREF_6) [^11^](#_ENREF_11).

**Comorbidity adjustments**

A general comorbidity correction was applied to all estimates of YLD using microsimulation methods to create hypothetical populations for each age group, by gender, year and country. For each simulated person in this age, sex, year and country stratum, a ‘combined’ disability weight was estimated to capture the presence of health states as per a multiplicative method. The reduction in weighting between the original (surveyed) disability weight for cannabis dependence and the simulated version which adjusted for comorbidity was used as the comorbidity correction for cannabis dependence YLDs. The probabilities of having no, one or more non-fatal health states simultaneously were based on the prevalence estimates for each health state. Analyses of the MEPS data that we also used to derive severity distributions for the main disabling conditions indicated that independent comorbidity (assuming no correlation) was far more decisive in predicting levels of comorbid disability than dependent comorbidity. For pragmatic reasons, i.e. the difficulty in finding information on all potential correlations and the large additional computational burden, we decided to take only independent comorbidity into account. For each hypothetical person in the microsimulation a combined disability weight was calculated between any comorbid health states using a multiplicative function and then reapportioned to each health state proportional to the sum of comorbid disability weights. The average ‘corrected’ disability for each health state was calculated in each age, sex, year and country stratum and the decrement compared to the original disability weight taken as the comorbidity correction for YLD.

**Covariate adjustment in DisMod-MR**

As the DisMod-MR modelling of cannabis dependence included estimates of both cannabis use and dependence, we used a ‘cannabis use’ study-level covariate to adjust cannabis use estimates downwards to an equivalent value if the study had captured cannabis dependence. Step 1 involved the DisMod modelling of regular cannabis use data. Analyses outside of DisMod-MR first adjusted estimates of past year cannabis use towards the desirable which were estimates of weekly cannabis use. This was based on a past year: weekly use ratio calculated outside of DisMod using a homogenous selection of prevalence estimates in the dataset. DisMod-MR was then used to model the regular (i.e. weekly) cannabis use prevalence data. Step 2 involved the DisMod-MR modelling of the cannabis dependence data. Analyses outside of DisMod-MR first adjusted estimates of past year prevalence towards the desirable which were estimates of point prevalence. This was based on a past year: point prevalence ratio calculated outside of DisMod using a homogenous selection of prevalence estimates in the dataset.

Figure 1a and b compare the DisMod-MR input prevalence estimates (which included estimates of both use and dependence) to the final DisMod MR output for males in 2010 from Australasia, a region for which we had a considerable amount of data available. The solid red line indicates the specified minimum age of onset of 13 years; the blue crosses represent the individual prevalence data points; the solid blue shows the final pooled prevalence output. Figure 1a plots the data points (blue crosses) before they were adjusted by the cannabis use covariate and Figure 1b plots them after they were adjusted i.e. the difference between the two figures represents the adjustment made by the covariate.

Overall, cannabis use data points in the entire models were adjusted downwards by a factor of 5.3 (4.3-6.5) to reflect their corresponding value if the studies had captured cannabis dependence.

**Figure 1. Prevalence before and after covariate adjustment for males from Australasia, 2010**

| **Figure 1a.Estimates before covariate adjustment** | **Figure b. Estimates after covariate adjustment** |
| --- | --- |
| 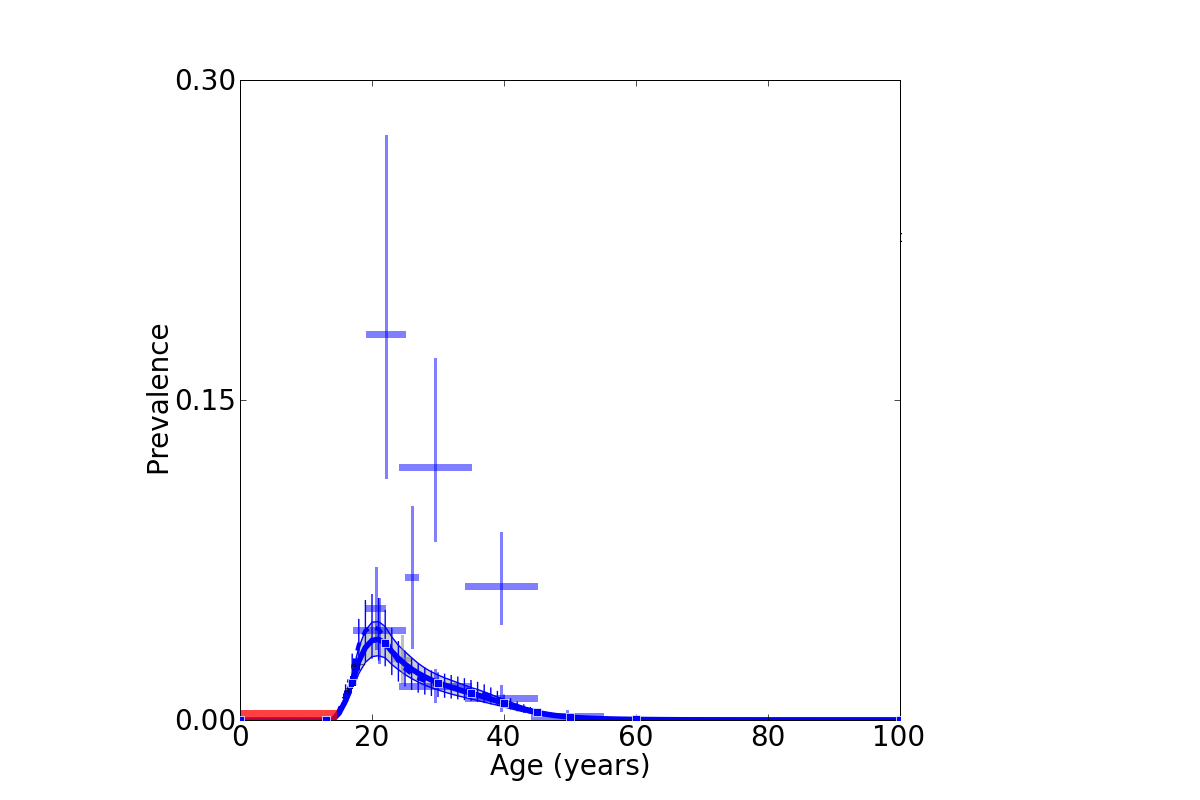 | 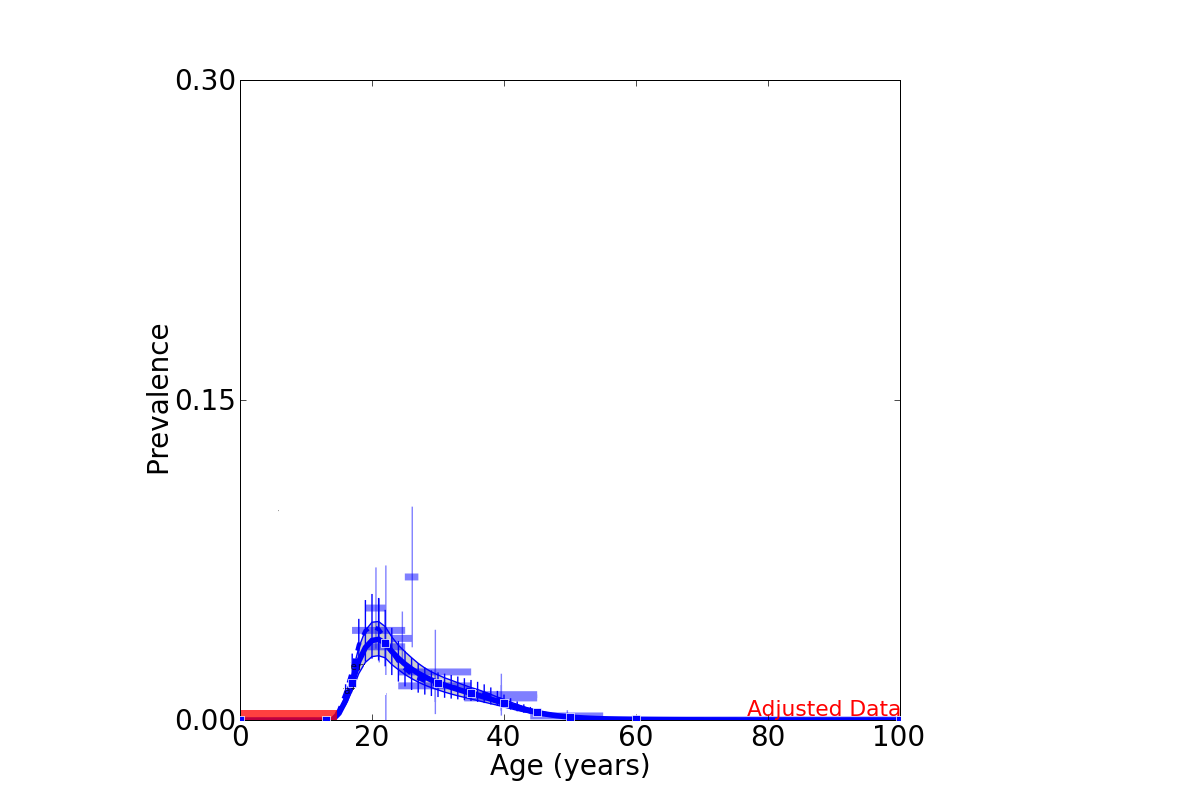 |
