# Supplementary material for: The Global Epidemiology and Contribution of Cannabis Use and Dependence to the Global Burden of Disease: Results from the GBD 2010 Study
Source: PLoS One. 2013 Oct 24;8(10):e76635. doi: 10.1371/journal.pone.0076635 (PMC3811989; doi:10.1371/journal.pone.0076635)
Supplement: Text S2 — Comparative risk assessment: cannabis use as a risk factor for schizophrenia. (DOCX) [file pone.0076635.s008.docx]

**Text S2: Comparative risk assessment: cannabis use as a risk factor for schizophrenia.**

We considered several ways in which cannabis and schizophrenia may be linked: Model 1) a model that assumed greater disorder severity among those using cannabis regularly who have already developed the disorder; Model 2) a model that assumed the association reflects earlier onset of schizophrenia among those who would have developed it anyway; Model 3) a model that assumed reduced remission from schizophrenia once it has developed; and Model 4) a model that assumed increased incidence of schizophrenia.

After consideration, approaches 3 and 4 were excluded from core GBD analyses because of the lack of data needed to systematically quantify the relationship across different studies while also accounting for confounding variables. Approaches 1 and 2 were also deemed more plausible on the basis of the literature[^12^](#_ENREF_12).

Data on regular (weekly or more frequent) cannabis use in the past year

We found seven studies reporting prevalence of weekly or more frequent cannabis use in the past year, from 15 countries and five GBD world regions; and 80 studies on the prevalence of past-year cannabis use, from 82 countries and 17 GBD world regions.

The epidemiological data available for regular cannabis use were modelled using an age-integrating Bayesian hierarchical model (DisMod-MR). Estimates of prevalence were derived separately for 21 world regions, males and females, 5 year age groups. We assumed zero incidence and prevalence of regular cannabis use before age 13 as this led to the most plausible fit to the data. A study-level covariate was used to crosswalk measurements of past year cannabis use to the preferred recall period of weekly cannabis use. Prevalence from past year use were 3.79 (3.48-4.13) times higher than estimates of weekly cannabis use and were adjusted downwards accordingly.

The final models used are presented in the figure below.

**Figure: Estimated regional distribution of regular (weekly or more frequent) cannabis use by sex, 2010**


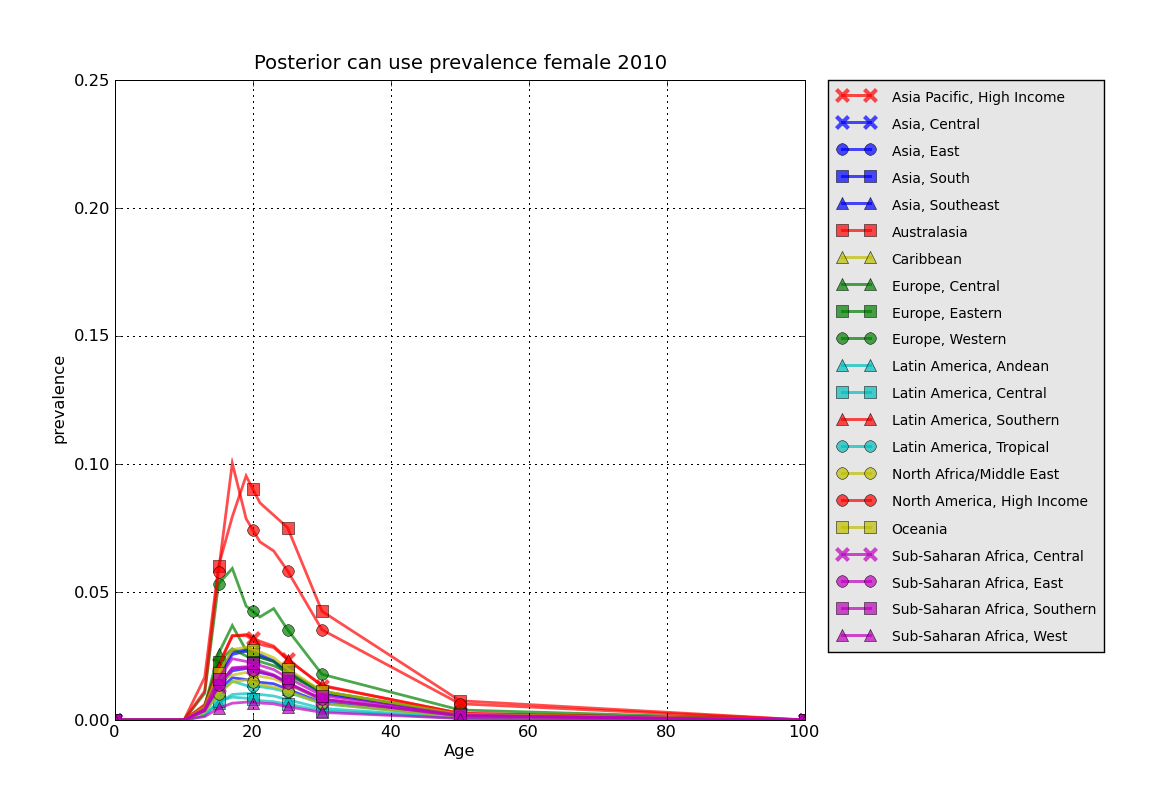


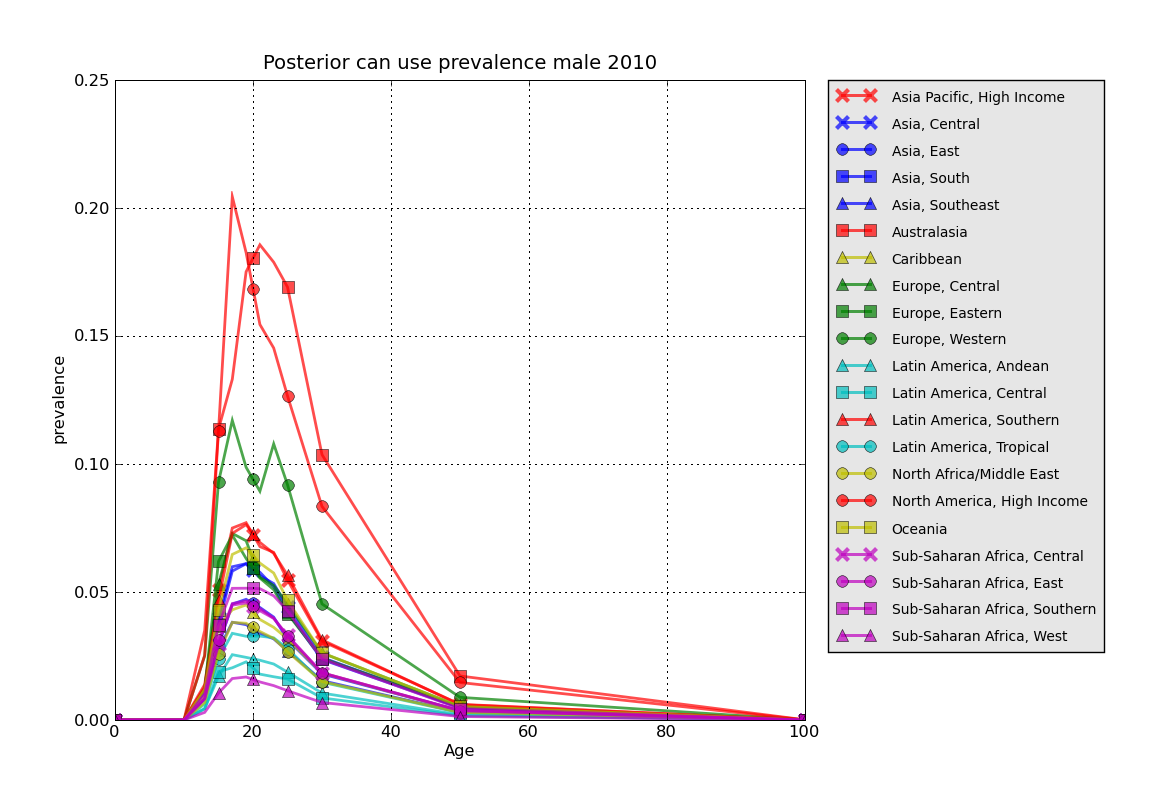


Modelling Earlier age of onset

The effect of cannabis use on schizophrenia was modelled via two pathways: the first is by causing the average age of onset to be earlier than with no cannabis use, and the second is by increasing the severity of schizophrenia. To account for the effects of the first pathway, we calculated the counterfactual average duration of schizophrenia across all ages under a scenario of no cannabis use, and compared this to the currently observed duration. To determine the counterfactual average duration of schizophrenia, we shifted incident cases of schizophrenia who use cannabis to be 2.70 (95% CIs: 1.96-3.43) years later based on a systematic meta-analysis by Large et al[^13^](#_ENREF_13).

We used the estimates of cannabis use by age (in single years), sex, country and year described above, assuming that the prevalence of regular cannabis was the same among individuals with schizophrenia and individuals without schizophrenia. Estimates of the number of incident cases and the corresponding duration of schizophrenia by age, sex, country and year were based on the DisMod model for Schizophrenia. The value of one minus the ratio of the counterfactual to the observed duration is an estimate of the PAF of schizophrenia due to the effect of regular cannabis use on age of onset.

**Modelling Increased severity of schizophrenia**

To calculate the burden associated with second pathway of shifting severity, we used the odds ratio from Foti et al[^14^](#_ENREF_14) of psychotic symptoms of 1.64 (95% CIs: 1.12-2.34) in people with schizophrenia who regularly use cannabis as opposed to those who do not. We converted the ORs to their RR equivalents based on the prevalence of exposure to regular cannabis use and the outcome of percent of time with psychosis (as opposed to residual state). The percent of time spent in acute psychosis was 63% (38%-82%) based on a meta-analysis of 6 studies covering 5 GBD world regions. We used the linear relationship between the estimated change in disability weight (based on the proportion of time spent in a psychotic state) and the prevalence of regular cannabis use to calculate the percent of schizophrenia disability attributable to regular cannabis use.

**References**

1. Calabria B, Degenhardt L, Briegleb C, Vos T, Hall W, Lynskey M, et al. Systematic reviews of prospective studies investigating “remission” from amphetamine, cannabis, cocaine and opioid dependence. *Addictive Behaviors* 2010;35:741-49.

2. Calabria B, Degenhardt L, Hall W, Lynskey M. Does cannabis use increase the risk of death? Systematic review of epidemiological evidence on adverse effects of cannabis use. *Drug and Alcohol Review* 2010;29:318-30.

3. Calabria B, Degenhardt L, Nelson P, Bucello C, Hall W, Lynskey M, et al. What do we know about the extent of cannabis use and dependence? Results of a global systematic review. NDARC Technical Report No. 307. Sydney: National Drug and Alcohol Research Centre, University of NSW, 2010.

4. Degenhardt L, Bucello C, Calabria B, Nelson P, Roberts A, Hall W, et al. What data are available on the extent of illicit drug use and dependence globally? Results of four systematic reviews. *Drug & Alcohol Dependence* 2011;117:85-101.

5. Salomon JA, Vos T, Hogan DR, Gagnon M, Naghavi M, Mokdad A, et al. Common values in assessing health outcomes from disease and injury: disability weights measurement study for the Global Burden of Disease Study 2010. *Lancet* 2012;380(9859):2129-43.

6. Murray CJL, Vos T, Lozano R, Naghavi M, Flaxman AD, Michaud C, et al. Disability-adjusted life years (DALYs) for 291 diseases and injuries in 21 regions, 1990-2010: a systematic analysis for the Global Burden of Disease Study 2010. *Lancet* 2012;380(9859):2197-223.

7. Vos T, Flaxman AD, Naghavi M, Lozano R, Michaud C, Ezzati M, et al. Years lived with disability (YLDs) for 1160 sequelae of 289 diseases and injuries 1990-2010: a systematic analysis for the Global Burden of Disease Study 2010. *Lancet* 2012;380(9859):2163-96.

8. Stroup DF, Berlin JA, Morton SC, Olkin I, Williamson GD, Rennie D, et al. Meta-analysis of observational studies in epidemiology: A proposal for reporting. *JAMA* 2000;283(15):2008-12.

9. Grant BF, Moore T, Kaplan K. Source and accuracy statement: Wave 1 National Epidemiologic Survey on Alcohol and Related Conditions (NESARC). Bethesda, MD: National Institute on Alcohol Abuse and Alcoholism, 2003.

10. Ware Jr JE, Kosinski M, Keller SD. A 12-Item Short-Form Health Survey: construction of scales and preliminary tests of reliability and validity. *Medical care* 1996;34(3):220-33.

11. Salomon JA. New disability weights for the global burden of disease. *Bulletin of the World Health Organization* 2010;88(12):879-79.

12. Degenhardt L, Hall W, Lynskey M, McGrath J, McLaren J, Calabria B, et al. Should we make burden of disease estimates for cannabis use as a risk factor for psychosis? *PLoS Medicine* 2009;6(9):Doi:10.1371/journal.pmed.1000133.

13. Large M, Sharma S, Compton MT, Slade T, Nielssen O. Cannabis Use and Earlier Onset of Psychosis: A Systematic Meta-analysis. *Arch Gen Psychiatry* 2011:archgenpsychiatry.2011.5.

14. Foti DJ, Kotov R, Guey LT, Bromet EJ. Cannabis use and the course of schizophrenia: 10-year follow-up after first hospitalization. *American Journal of Psychiatry* 2010;167(8):987-93.
